# Pharmacology of Natural Volatiles and Essential Oils in Food, Therapy, and Disease Prophylaxis

**DOI:** 10.3389/fphar.2021.740302

**Published:** 2021-10-21

**Authors:** Nicholas John Sadgrove, Guillermo Federico Padilla-González, Olga Leuner, Ingrid Melnikovova, Eloy Fernandez-Cusimamani

**Affiliations:** ^1^ Jodrell Science Laboratory, Royal Botanic Gardens, Kew, Richmond, United Kingdom; ^2^ Department of Crop Sciences and Agroforestry, Faculty of Tropical AgriSciences, Czech University of Life Sciences Prague, Prague, Czech Republic

**Keywords:** pharmacokinetics, pharmacodynamics, pathogen, antimicrobial, anti-inflammatory, gas chromatography, headspace, aromatherapy

## Abstract

This commentary critically examines the modern paradigm of natural volatiles in ‘medical aromatherapy’, first by explaining the semantics of natural volatiles in health, then by addressing chemophenetic challenges to authenticity or reproducibility, and finally by elaborating on pharmacokinetic and pharmacodynamic processes in food, therapy, and disease prophylaxis. Research over the last 50 years has generated substantial knowledge of the chemical diversity of volatiles, and their strengths and weaknesses as antimicrobial agents. However, due to modest *in vitro* outcomes, the emphasis has shifted toward the ability to synergise or potentiate non-volatile natural or pharmaceutical drugs, and to modulate gene expression by binding to the lipophilic domain of mammalian cell receptors. Because essential oils and natural volatiles are small and lipophilic, they demonstrate high skin penetrating abilities when suitably encapsulated, or if derived from a dietary item they bioaccumulate in fatty tissues in the body. In the skin or body, they may synergise or drive *de novo* therapeutic outcomes that range from anti-inflammatory effects through to insulin sensitisation, dermal rejuvenation, keratinocyte migration, upregulation of hair follicle bulb stem cells or complementation of anti-cancer therapies. Taking all this into consideration, volatile organic compounds should be examined as candidates for prophylaxis of cardiovascular disease. Considering the modern understanding of biology, the science of natural volatiles may need to be revisited in the context of health and nutrition.

## Introduction: Medical Aromatherapy

The modern culture of aesthetic aromas and volatile organic compounds in human health is polarised by controversiality, with the prevailing criticism being the lack of scientific credibility. Yet there is ample scientific evidence of modest to noteworthy biological effects from aromatic plant-based cosmetics, nutraceuticals, and therapies. Unfortunately, the subjectivity in the aromatherapeutic industry and the credulity of participants has attracted much negative attention.

Consequently, the Cambridge English Dictionary defines aromatherapy as …

‘the treatment of worry or nervousness, or medical conditions that are not serious, by rubbing pleasant-smelling natural substances into the skin or breathing in their smell’

As exemplified above, the prevailing view in western societies is that aromatherapy is limited to either massaging with essential oils or the inhalation of plant-derived volatile organic compounds to achieve mood altering effects. However, a search of clinicaltrials.gov provides an alternative overview of the applications of essential oils: out of hundreds of studies less than a quarter were dedicated to mood altering effects, i.e., a search of ‘essential oils’ gave several results related to mouth washes, throat gargles, pain management, antiseptic applications, facial acne and muscle cramps.

Definitions of aromatherapy, as explained in dictionaries, encyclopaedias or portrayed in memes, do not acknowledge the diversity of techniques that are corroborated by empirical science. For example, the essential oil of *Cordia verbenacea* A. DC administered to rats, systemically or orally, confers noteworthy anti-inflammatory effects (Medeiros et al., 2007). It is marketed in Brazil as an active ingredient in the product Acheflan which is applied topically. The positive effects of Acheflan are achieved *via* the aroma molecules, *E*-caryophyllene and α-humulene (Fernandes et al., 2007).

The problem of correctly defining aromatherapy is also strained by a lack of cultural inclusiveness in the current definition (Sadgrove N. J., 2020). Under the umbrella of aromatherapy, aromatic extracts have been used in indigenous African cultures to alleviate foot odours (Hulley et al., 2019) and in steam/smoke inhalation therapies (Khumalo et al., 2019). Similarly, in Australian Aboriginal cultures aromatic plants are used successfully to treat fungal infections in the form of fat extracts (Sadgrove et al., 2011; Sadgrove and Jones, 2014b) and in smoke fumigation applications (Sadgrove and Jones, 2013; Sadgrove et al., 2014).

Research on volatile organic compounds is starting to convey that potentiation of other products is occurring more often than realised, such as in antimicrobial outcomes (Mikulášová et al., 2016) or other mainstream medicines. Immunomodulatory effects are also being observed in relation to cytokine release (Anastasiou and Buchbauer, 2017), T-cell proliferation (Anastasiou and Buchbauer, 2017), agonism of membrane receptors (toll-like (Amiresmaeili et al., 2018)) or nuclear receptors (PPAR (Goto et al., 2010)) and reduced mast cell degranulation (Anastasiou and Buchbauer, 2017).

With growing scientific validation there is a widening polarization of the schools of aromatherapeutic practice, with one side aligned to the supernatural, and the other on the more traditional medical ethos. However, a distinction clearly needs to be made. The author Kurt Schnaubelt successfully made this distinction by the use of the elaborated term ‘medical aromatherapy’ (Schnaubelt, 1999) to imply a more objective approach to therapy with essential oils and natural volatiles.

Thus, ‘medical aromatherapy’ can be defined as …

‘the objective of achieving a health benefit from topical application, oral administration, or inhalation of a natural product mixture that includes at least one “active” or “coactive” volatile organic compound

In this definition it is explained that medical aromatherapy can also be achieved by using raw aromatic plants, as crushed leaves or extracts, to achieve therapeutic effects (Sadgrove, 2020b), as an alternative to hydrodistilled essential oils. Although the two are not mutually exclusive, medical aromatherapy practitioners are not restricted to the use of essential oils because volatile organic compounds are also present in aromatic extracts, such as supercritical CO_2_ extracts of aromatic leaves (Damjanovic et al., 2006; Wenqiang et al., 2007), or aromatic fat extracts familiar to the French practice of enfleurage. In this scenario the volatile compounds are not necessarily the sole driver of efficacy because plant organs and extracts contain other families of metabolites, i.e., the chemical diversity of whole aromatic plants includes volatile and non-volatile ingredients that may achieve combined effects (potentiation, synergism, or additive) in the context of medical aromatherapy (Langat et al., 2021; Nsangou et al., 2021). In this latter hypothetical, volatile organic compounds are ‘coactive’, meaning that they contribute to efficacy but are not the only driver of efficacy.

Aromatic plants are rich in volatile organic compounds that can be distilled to produce essential oils, but it is not correct to call them essential oils prior to separation by distillation, i.e., aromatic plants do not contain essential oils, in the same way that milk does not contain cheese or wheat does not contain bread. According to the modern definition of an essential oil provided by the International Standards Organisation, a single organic compound, such as limonene, is not an essential oil, it is a volatile organic compound that is a common ingredient in an essential oil: it is an ‘essential oil component’, to use the expression coined by Adams (2007) to make this important distinction. Essential oils are mixtures of volatile organic compounds that have been separated by distillation from aromatic species, including bryophytes, such as liverworts (Asakawa and Ludwiczuk, 2013), and higher plants.

## The Current Research Paradigm

In recent decades, contingent with the increasing convenience of chemical analysis of volatiles, there has been an unprecedented number of publications reporting the chemistry of essential oils from the world’s flora. This large body of data was born from the collective of laboratories equipped with the universal mass spectral detector at the end of a gas chromatography column (GC-MS) (Sadgrove NJ., 2020; Sadgrove N. J. et al., 2020). However, world experts in the field of natural volatiles and essential oils are now unanimously encouraging a shift of emphasis away from mere chemical reports.

For some time the superfluity of chemical reports, and competitiveness in publishing, were overcome by including results of *in vitro* antibacterial testing, to add value to the dataset (Guimarães et al., 2019). These *in vitro* studies use micro-titre plate broth-dilution assays of whole essential oils to derive minimum inhibition concentration (MIC) values against pathogenic or model bacteria (Sadgrove and Jones, 2015; Van Vuuren and Holl, 2017). While such information is valuable, a pattern has emerged that makes further work predictable, and generally the MIC values are not regarded as pharmacologically interesting from a commercial perspective (Sadgrove and Jones, 2019). Furthermore, these methods omit the antimicrobial activity of volatile organic compounds that are in the vapour phase, which is more inhibitory compared to the liquid phase. The methods for determination of the antibacterial effect of volatile agents that are simultaneously in the liquid and vapor phase were developed recently (Houdkova et al., 2017; Houdkova et al., 2021). Nevertheless, with no consideration to the vapour phases of volatile organic compounds, high concentrations (0.1–20 mg.mL^−1^) are required to achieve antibacterial effects of essential oils. If some bactericidal essential oil components were to be present in human plasma at concentrations high enough to have antibacterial effects, they will enact toxic and potentially lethal effects to the person. Hence, antibacterial effects of essential oils are best represented in different contexts, in topical applications to treat odours and fungal infections, in sterilizing skin or surfaces, laundry and so forth (Jones and Sadgrove, 2015; Van Vuuren and Holl, 2017). Furthermore, volatile organic compounds present in edible aromatic species can influence the gut microbiota and attenuate fermentation or bacterial overgrowth in gastrointestinal pathologies (Li et al., 2018). High gastrointestinal concentrations will not lead to high systemic concentrations because metabolism of volatile organic compounds is generally efficient. Because *in vitro* MIC values for contact inhibition are not possible, it is better to consider the apparent immunomodulatory and gene regulatory effects (Sadgrove and Jones, 2019).

Unfortunately, complementary studies that translate phytochemical knowledge into pharmacological serendipity for wider human benefit are not being pursued outside of just a few laboratories. Pioneering new ways to appreciate essential oils and volatiles require pushing the boundaries of encapsulation methodology, extraction technology, food preservation, knowledge of synergistic activity or potentiation in the plight of resistance mechanisms in pathogenic bacteria and their effects in human physiology. Thus, since the dawn of the 21st century, particularly in the last few years, numerous research groups have shifted emphasis towards utilising the phytochemical information that has been amassed hitherto.

## A Brief History of Essential Oils

Even without the integrated efforts of scientists, human interest in volatile organic compounds and distilled essential oils will continue to be independently motivated. This can be partly explained by the aesthetic experience in aroma, which reinvigorates the cultural and symbolic significance. Humans have utilised the aromatic principle of plants since before recorded history and contingent with the development of modern-type hydrodistillation technology essential oils *per se* were ‘invented’ in the 1200 AD by Arabic pioneers (Bauer and Garbe, 1985; Sadgrove and Jones, 2015). However, long before modern hydrodistillation methods low quality essential oils were captured using a primitive apparatus invented by the Persians, that was better for making floral waters. Remains of the Persian terracotta distillation apparatus are estimated to be approx. 3500 B.C. Something similar was also used by the ancient Egyptians, who packed the outlet pipe with rags to create a type of condenser that captured floral waters and some of the essential oil, which could be collected from the rags by compression.

The sophistication of modern technology means that essential oils can be produced in mass by hydrodistillation (plant material in boiling water), steam distillation (plant material placed in path of steam) and microwave assisted steam distillation. In this regard, the modern definition of an essential oil as dictated by the International Standards Organisation is a …

“product obtained from natural raw material, either by distillation with water and steam, or from the epicarp of citrus fruits by mechanical processing, or by dry distillation” (Schnaubelt, 1999; ISO, 2015).

The etymological background of the term ‘essential oil’ is in the Latin expression ‘*quinta essentia’* which literally means 5^th^ element. The essential oil was referred to as the soul or spirit of the plant, which is strongly related to the etymology of the term ‘spirits’ to mean alcohol or liqueur (Sell, 2010). Essential oils should never be referred to as ‘extracts’ or ‘extracted’, because they can only be produced by evaporation; essential oils are actually ‘exorcized’ from the plant, not extracted, which would require the use of solvents or mechanical pressing. The only exception to this is from the epicarp of citrus fruits, but this is due to the inertia of pre-established rural language.

Sometimes a single aromatic plant species can be divided according to distinct chemical groups called chemotypes. In rare cases, one species can be divided into as many as 10 or more chemotypes that have completely different chemical profiles (Sadgrove and Jones, 2014a). While chemotypes tend to be highly consistent in terms of chemistry (i.e., borneol type always has borneol), the chemistry can also change in response to environmental factors and seasonal variation, causing an effect called phenotypic plasticity (Sadgrove NJ., 2020). Phenotypic plasticity can create chemotypes within species, or it can occur on a spectrum, which involves many entities with chemistry that overlap between chemotypes. In the Australian flora, volatile compounds can appear or disappear from the chemical profile in response to wet and dry cycles of weather (Sadgrove NJ., 2020) or other factors.

## Chemophenetics of Essential Oils and Solvent Extracts

It is often the case that the chemical profiles within species are ‘flamboyant’ (Sadgrove NJ., 2020; Sadgrove NJ. et al., 2020), i.e., highly variable, which can be caused by abiotic stressors that change expression patterns of volatiles, known as ‘phenoplasticity’, as mentioned above. In these cases, a known plant species is not guaranteed to deliver the same essential oil chemical profile. It is therefore important to be cognisant of chemical variation that could be caused by chemotypes or environmental factors, particularly in the context of health claims for the essential oil components. For example, two chemotypes of oregano are known, the thymol type and the carvacrol type (Bedini et al., 2021). Hence, it is important to be aware of these differences if used in therapeutic or prebiotic applications.

The discipline that examines the potential chemical differences within species is formally known as ‘chemophenetics’. This subject title is used today as a replacement for the old term ‘chemotaxonomy’ (Zidorn, 2019). The new name was necessary to avoid criticism because in classic chemotaxonomy it was imagined that chemical profiles could be used to fingerprint taxa with high reproducibility, but phenoplasticity and the existence of chemotypes within taxa antagonised reproducibility.

In the last 5 years chemophenetic research of volatile organic compounds has started to utilise solvent extracts, rather than hydrodistilled essential oils. This is both convenient and creates more detailed information. While hydrodistillation requires masses of leaves, energy input, time, and effort to produce essential oils, solvent extraction requires a small leaf and a small volume of solvent (DCM, Hexane). This method was used in a chemophenetic study of heterogeneous species aggregates in *Eucalyptus* (Collins et al., 2018), *Phebalium nottii* (Sadgrove N. J. et al., 2020) and *Eremophila* (Sadgrove et al., 2021), and in the former two the leaf samples were taken from herbarium voucher specimens. In the case of *Eucalyptus*, the sesquiterpene diol cryptomeridiol does not survive hydrodistillation and eliminates a hydroxyl group to randomly produce three eudesmols, either alpha (α-), beta (β-), or gamma (γ-). By using solvent extraction instead, cryptomeridiol is detected (Collins et al., 2018). In the pink flowered *Phebalium nottii* complex, putative new species were often in significant agreement with semi-volatile coumarins that have vapour pressures too low to be produced in hydrodistillation. The semi-volatile coumarins were easily detected by GC-MS if the column temperature was raised to 280–300°C and held for 20 min (Sadgrove N. J. et al., 2020). Species in *Eremophila* also express semi-volatiles that may have significance in taxonomic studies because of a reduced susceptibility to the effects of phenoplasticity (Sadgrove et al., 2021). In this latter study it was realized that the effects of phenoplasticity from contemporary weather changes, such as droughts or excessive wet periods, are more dramatic in leaf material than in timber. It was suggested that chemophenetic studies may yield more reproducible data if the timber volatiles are studied, rather than leaves.

## Factors Affecting Essential Oil Collection

The amount of an essential oil in a species, as determined by the ‘yield’ from hydrodistillation, can vary considerably. The phenylpropanoid dominated essential oil from clove can yield as high as 7.4–11.5% g.g^−1^ from dried cloves or 1.2% if fresh (Wenqiang et al., 2007; Murni et al., 2016). This contrasts with the oleo-resin made by supercritical CO_2_ extraction which yields 15–20% g.g^−1^ from dried clove buds, but this higher yield is related to the presence of non-volatile substances such as cuticular waxes (Wenqiang et al., 2007). Some Australian species also demonstrate very high yields, such as the monoterpene-rich isomenthone, or karahanenone diploid chemotypes of *Eremophila longifolia* (Smith et al., 2010; Sadgrove et al., 2011; Sadgrove and Jones, 2014a) yielding from 2–10% g.g^−1^ of wet leaves, which varies yearly according to drought effects. A similar phenylpropanoid-rich safrole/methyl eugenol chemotype is known from the country’s far west. Another high yielding genus includes the sesquiterpene-rich heterogeneous species aggregates of *Prostanthera* sp. aff. *ovalifolia* and *P*. sp. aff. *rotundifolia* (Sadgrove et al., 2015), which invariably give 1–2% g.g^−1^ from fresh leaves.

Low yields can make it difficult to produce an essential oil, which makes them very expensive in the market, such as rose essential oil from *Rosa damascena* Mill., which yields only 0.03% g.g^−1^ from fresh rose petals after steam or hydrodistillation (Dobreva et al., 2011). In this regard, careful temperature and hydrosol modulation is required to ensure successful condensation and collection, respectively. Gases need to be sufficiently cooled by the condenser to adequately capture the volatiles, returning them to the liquid phase. They are then pooled in a chamber that is less than 30°C, to prevent re-evaporation. The water phase, called the hydrosol, needs to be minimised because part of the oil is dissolved there. Although volatile organic compounds are only slightly aqueously soluble, high volumes of hydrosol and small quantities of essential oil can make the difference between successful collection and failure. Some distillers use cohobation, which is the process of returning the hydrosol to the boiler to ensure recovery of dissolved components. An even better design is the Clevenger apparatus, a near century old design (Clevenger, 1928) that captures only a small amount of hydrosol and returns the rest to the boiler in real time.

Acids are generally not volatile enough to be evaporated in hydrodistillation unless they are extremely small, but their small size means they are mostly dissolved into the hydrosol and phase separated from the oil, such as in the case of the boswellic acids of *Boswellia serrata* (Raman and Gaikar, 2003). Esterification of acids makes them more volatile. Hence, esters of acids are detected in essential oils, such as the C19-norditerpene ‘gratissihalimanoic ester’ from *Croton gratissimus* (Sadgrove et al., 2019). As previously mentioned, diterpene dominated essential oils are uncommon. It is also rare to find benzoic acid derivatives, such as *p*-methoxycinnamate identified in the essential oil of *Eriostemon obovalis* (now *Philotheca obovalis*) by the late Erich Lassak in 1974 (Lassak and Southwell, 1974).

## Essential Oils in Medical Aromatherapy

The European Pharmacopoeia lists 28 essential oils, defining them as safe (Pauli and Schilcher, 2010). Unfortunately there are also many essential oils that have potential in human health but are rejected on the basis of poorly performed safety assays, such as thujone-rich oils (Németh and Nguyen, 2020). Essential oils and their components are pharmacologically versatile. As previously mentioned, they are lipophilic, which enables them to absorb into and interact with prokaryotic and eukaryotic cell membranes. They also affect neuronal and muscle ion channels, neurotransmitter receptors, G-protein coupled (odorant) receptors, second messenger systems and enzymes (Bowles, 2003; Buchbauer, 2010).

### Pharmacokinetics of Volatile Organic Compounds

For any organic compound to be volatile it must have low molar mass and low polarity. Low polarity is also expressed as lipophilicity (fat solubility). As a progression, volatile organic compounds are dissolved into and transverse human skin layers (Cal, 2006), due to the phospholipid membranes of squamous cells and the phospholipid bilayer of the extracellular matrix. Lipophilic compounds with moderate polar head space, such as by having a keto or hydroxyl group, travel through the dermis faster than carbures (hydrocarbons), however even α-pinene can follow the transdermal route, albeit fluxing at a slower rate than components such as linalool or terpinen-4-ol (Cal, 2006). Nevertheless, because essential oil components are penetration enhancers of other drugs (Okabe et al., 1990; Chen et al., 2016), it is feasible that they are also enhancers of other components in an essential oil, meaning that carbures in combination with moderately polar components (i.e., terpinene-4-ol or linalool) will have more efficient transdermal penetration. Unfortunately, not much is known about the differences of absorption with whole essential oils compared to individual components.

Hence, topical application of essential oil components and transdermal penetration is more efficient than expected by non-specialists, but an encapsulation vehicle, such as a pressed oil (i.e., rosehip oil) is sometimes necessary to augment this effect, particularly to slow the rate of evaporation of the essential oil from the skin. For example, 97% of topical linalool was evaporated if applied with ethanol onto the skin (Green, 2007), but if mixed with a fixed oil ‘carrier’ most of it is absorbed (Jäger et al., 1992). Furthermore, East Indian Sandalwood essential oil (*Santalum album*) was topically applied onto candidates who wore a face mask to prevent inhalation of the aroma and resulted in statistically significant physiological changes, such as blood pressure, pulse rate and ‘alertness’ compared to the control (Hongratanaworakit et al., 2004). Inhaled essential oils can also become systemic and lead to changes in metabolic pathways associated with anxiety related behaviour, which has been demonstrated to occur in rats (Wu et al., 2012).

Topically applied, ingested or inhaled essential oils, or aromatic extracts, release components into the body that rapidly ‘sink’ into fat tissue, while some components are transported around the body in the vehicle of blood albumin. Compounds with keto groups (carbonyls) bind to blood albumin and are circulated throughout the body but are thereafter eliminated in metabolism or sunk into adipose tissue or the phospholipid membranes of some cells, like keratinocytes. Components that are *trans*-dermally absorbed (via lungs or skin) enter capillaries and the blood stream, where they are detected within 20 min and for as long as 90 min (Jäger et al., 1992) before sinking and/or eliminated in metabolism. Lipophilic compounds cross the blood brain barrier, and can create psychoactive effects, such as the phenylpropanoid elemicin (Beyer et al., 2006), the terpene incensole acetate (Moussaieff et al., 2008) or the phytocannabinoids (Griffin et al., 1999).

The transdermal route greatly slows the metabolism of compounds by avoidance of the ‘first pass’ effect that occurs in digestion of orally administered matter, where metabolites entering portal circulation from the intestines are circulated directly to the liver (Sadgrove and Jones, 2019). However, in some cases, the oral route to the absorption of volatile organic compounds is more convenient. For example, although *d* -limonene does not have a keto group, plasma levels reached as high as 1.65 μM with lemonade drinking and over the course of 4 weeks accumulated in adipose tissues to levels nearly 200 fold greater than maximum plasma concentration (Miller et al., 2010). Alternatively, via the oral route linalyl acetate is immediately converted into linalool in the digestive process (Nölder et al., 2011), and linalool concentrations peak in blood plasma at 1915 ng ml^−1^ (Shi et al., 2016). Thus, in the case of linalyl acetate, topical application is better, i.e., topical application of lavender essential oil to a human abdomen resulted in maximum plasma concentrations of >250 ng ml^−1^ essential oil, made up by 100 ng ml^−1^ linalool and 121 ng ml^−1^ linalyl acetate (Jäger et al., 1992). Linalool accumulates in organs and fat at concentrations many folds higher than peak plasma concentrations (Nölder et al., 2011). Similarly, in the ‘Karoo’ of South Africa a lamb that forages on *Pentzia incana* (Thunb.) Kuntze reputably acquires an artemisia flavour to its meat, known as the ‘Karoo lamb’, which is a consequence of volatile organic compounds accumulating in its adipose tissues (Hulley et al., 2018).

Essential oil components can also have prooxidant effects that are a negative consequence of higher than safe levels (Bakkali et al., 2008). This is of relevance to phenylpropanoids and other phenolics that demonstrate pronounced *in vitro* radical scavenging abilities. As previously mentioned, lipophilic compounds dissolve into the phospholipid walls of human cells. The concentration determines if a positive or negative effect occurs, wherein a wide concentration range for positive therapeutic effects is available. Volatile organic compounds increase the permeability of phospholipid membranes, not just in cell walls but also in the walls around organelles. Permeabilization of the mitochondrial membrane can potentially interfere with the electron transport chain, leading to the upregulation of radical oxygen species that oxidise cellular contents. If phenolic compounds are present, their oxidation will generate significantly more reactive species (Bakkali et al., 2008). However, studies that report on prooxidant effects are still describing concentrations that are high, such as 30–90 μg ml^−1^ (200–600 μM) of carvacrol (Liang and Lu, 2012). While such concentrations may seem unrealistic, they are frequently reported as peak plasma concentrations in mice studies. For example, a pharmacokinetic study of borneol and menthol demonstrated peak plasma concentrations of 20 and 70 μg ml^−1^ respectively, which were metabolised in one and 3 hours respectively (Xu et al., 2011).

In considering pharmacokinetic studies wholistically, plasma concentrations peak instantly with intravenous administration, or after oral administration anywhere from 20 min to 3 h. The peak plasma concentrations are dependent upon dose, but *in vivo* rat models have demonstrated as high as 300 μg ml^−1^ in plasma, present mostly bound to plasma proteins (Dawidowics and Dybowski, 2014). Peak plasma concentrations are usually lower than tissue plasma concentrations, but tissue plasma concentrations are usually not measured in studies.

Essential oil components are either metabolised or absorbed into adipose tissues and organs, lowering plasma levels to baseline within 1–3 h for low doses, but at high doses plasma concentrations are maintained for several days as the components are buffered from the body’s tissues. Hence, during metabolism they are slowly removed from adipose tissue and the organs, which is highest in fat, followed by the liver, then kidneys and lowest in cerebrospinal fluids and brain. After 24–72 h after a single dose the components are still present in adipose tissue, where they persist for some time. This is corroborated by evidence presented in animal studies (Serrano et al., 2007).

### Metabolism and Safety of Volatile Organic Compounds

In metabolism, essential oil components are oxidised by phase 1 and 2 enzyme mediated reactions in the liver or other tissues (Zehetner et al., 2019) creating polar derivatives in phase 1, then sulphate, glutathione or glucuronide conjugates in phase 2. A list of metabolic derivatives of common essential oil components is given by Kohlert et al. (2000).

After the xenobiotics are metabolised by phase 1 or 2 processes they are then eliminated via urination or secreted into the bowel for microbial fermentation. For example, during a pharmacokinetic study of menthol, participants received an oral dose of >500 mg pure menthol, yet peak plasma concentrations did not exceed 160 ng ml^−1^ whereas menthol glucuronides were as high as 7 μg ml^−1^ (Valente et al., 2015). Although these glucuronides are created to facilitate the removal of menthol from the system, they may be recycled for therapeutic effects by deconjugation when in contact with the enzyme glucuronidase, which is upwardly expressed in the inflamed tissues of the body (Shimoi and Nakayama, 2005). This encourages us to think of the conjugated forms of essential oil components as quasi-bioavailable.

The phase 2 β-glucuronide metabolite is characterised by a glucuronide moiety O-linked to the xenobiotic (the compound). As previously mentioned, once these products reach this higher level of polarity, they have short half-lives because they are efficiently eliminated by the kidneys. However, high amounts of these polar conjugates can be dissolved in blood plasma and transported to any extracellular space in the body, reaching sites of infection or inflammation. Higher amount of β-glucuronidase alluded to above is common in cancers as well as inflamed tissues. When the glucuronide moiety is removed, the xenobiotic is returned to a much more hydrophobic intermediate, commonly a derivative that was formed in an earlier metabolic step, if not the pre-metabolised form. This causes the xenobiotic to lose solubility and accumulate on-site, potentially enacting biological effects locally (Sperker et al., 2001). Current research on the pharmacokinetics of natural products ignores this latter observation in the context of rational *in vivo* translation of *in vitro* outcomes (Sadgrove and Jones, 2019).

Glutathione conjugates are less commonly described as a metabolic product of essential oil components. When the glutathione conjugates were observed in earlier studies, they were thought to be non-enzymatic phase 2 reactions that were initiated by a phase 1 oxidation (Thompson et al., 1990). However, the understanding of glutathione S-transferases and their role in conjugation of glutathione to xenobiotics (Sheehan et al., 2001) changed this view. Several studies describe glutathione conjugates of essential oil components, such as cinnamaldehyde (Choi et al., 2001), pulegone (Lassila et al., 2016) and eugenol (Thompson et al., 1990), just to name a few. Conjugation by S-transferases typically creates an S-linked glutathione but in some cases N-linked conjugates are non-enzymatically formed, which can occur when furans form reactive aldehydes that react in a Schiff-base fashion with the free glutamyl amine on the glutathione reactant, which happens to menthofuran (Lassila et al., 2016). Essential oils are known to upregulate the expression of glutathione S-transferase in the liver (Banerjee et al., 1994; Abd El-Moneim et al., 2012), but minimal study has been dedicated to the P isoform that is upregulated in cancers (Tew et al., 2011). It is unclear if upregulation of glutathione S-transferase in cancers by essential oils is a positive or negative outcome because chemotherapeutic drugs are metabolised faster, which is a negative, but so are carcinogens, which is a positive. Furthermore, the biological effects of glutathione conjugates of essential oils have minimal research, but they should be examined in the context of cancers as part of the growing body of research dedicated to glutathione S-transferase prodrugs (Townsend and Tew, 2003). Finally, many xenobiotics are not conjugated to glutathione (Kohlert et al., 2000), and because there are minimal reports of this occurring in essential oil components, it may be considered less common.

While essential oil components are usually metabolised by both phase 1 and 2 processes in the liver, there is some evidence that more is ‘sunk’ into adipose tissues and organs than is eliminated, i.e., one study reported in humans that with 1 mg oral dose of thymol the peak plasma concentration reached 0.093 μg ml^−1^, but only about 16% was eliminated as thymol sulphate or glucuronide, suggesting accumulation in organs and fat (Kohlert et al., 2002).

Minimal studies are available to determine peak plasma or organ concentrations before toxic effects may be considered in people. A single study was found that examined the human maximum tolerance dose of *d* -limonene and quantities administered ranged from 0.5 to 12 g m^2^ orally. It was determined that the safe dose was 8 g m^2^ i.e., 12–16 g oral dose, which could be sustained for 11 months with no adverse effects. Despite such a high oral dose, the peak plasma concentrations were 2.12 μg ml^−1^ compared to tissue plasma concentration of 5.52 μg ml^−1^, and the major phase 1 metabolic products are perillic acid, perillic acid isomers, perillyl alcohol and limonene-diol derivatives. Hence, peak plasma concentrations of limonene combined with its oxidised forms were >14 μg ml^−1^ (Vigushin et al., 1998). Some biological roles of these metabolic products have been demonstrated, including immunomodulation, anti-inflammatory and antiproliferative effects against pancreatic cancer cells (da Silveira e Sá et al., 2013).

In mice there are several studies that push the limits in terms of safety. For example, intravenous and oral doses of 12.5 mg geraniol in mice produced peak plasma concentrations of approximately 250–300 μg ml^−1^ by both routes, which was metabolised or ‘sunk’ within 2 h. To test for toxicity the authors then administered a 10-fold higher concentration of 120 mg day^−1^ for 4 weeks and demonstrated no apparent toxic effects (Pavan et al., 2018). This indicates that mammals can experience plasma concentrations familiar to many of the *in vitro* studies, whilst remaining several orders of magnitude below possibly toxic concentrations. Evidently, toxicity is dependent on the functional groups of individual essential oil components, so they will need to be considered on an individual basis.

Unfortunately, there are limited studies that focus on the possible biological effects of the metabolic conjugates or phase 1 metabolites of essential oil components, i.e., phase 1 oxidised or phase 2 conjugated sulphate, glutathione or glucuronide forms. Although this has been visited to an extent in the cases of limonene metabolites (da Silveira e Sá et al., 2013), it is worth considering other leads in future studies.

Volatile organic compounds can also influence the expression and activity of cytochrome P450 enzymes and transferases involved in metabolism (Sadgrove and Jones, 2019), which can influence the nature of its own metabolism or the metabolism of other drugs in the system, either slowing down or speeding up the rate of metabolism and changing the drug’s half-life (Zehetner et al., 2019). There is a growing body of knowledge of the metabolism of essential oil components when administered in pure form (Zehetner et al., 2019), but less is known about component metabolism when ingested as part of parent plant material that also includes components that modulate cytochrome p450 (CYP) isozymes and change the rate of metabolism of specific components relative to others. In several cases plant material has CYP isozyme inhibitors that increase the peak plasma concentrations of the metabolite (Ashour et al., 2017). Furthermore, interactions of essential oil components with drugs should be taken into consideration if candidates use pharmaceuticals. A comprehensive guide to the safety of essential oils is given by Tisserand and Young (2013).

The ability of essential oil components to modulate CYP isozymes may in part be related to their affinity for the pregnane X receptor (Šadibolová et al., 2019), but recent evidence has not been conclusive. Nevertheless, a comprehensive summary of the enzymes that are modulated in relation to the essential oil component is provided by Zehetner et al. (2019), where induction, inhibition and metabolizing enzymes are listed.

### Additive, Synergistic, Antagonistic or Potentiator

It is common for studies to demonstrate interesting biological effects from crude extracts of plant organs, but to fail to identify an active ingredient after following a bioassay guided fractionation approach (Sadgrove and Jones, 2019). In admitting defeat, authors of these types of studies often speculate that synergism is responsible for irreproducibility of their earlier outcome. Unsurprisingly, it is indeed true that synergisms occur, but research has only recently started to explain these synergisms and essential oil components are repeatedly demonstrated to be significantly involved (Sadgrove N. J., 2020; Langat et al., 2021; Nsangou et al., 2021). However, during the fractionation process volatile components are often lost when removing solvents, making it difficult for researchers to recognise synergistic effects.

The effects of drug or compound combinations are classified according to the four categories, additive, synergistic, antagonistic or potentiator. In the context of antimicrobial studies, these categories are usually determined by testing combinations and calculating summed fractional inhibitory concentrations (ΣFIC). This is calculated using the minimum inhibition concentration (MIC) of individual components or essential oils and comparing to combinations. The MIC assay for antimicrobial testing is elucidated by Sadgrove and Jones (Sadgrove and Jones, 2015), but briefly the protocol uses a serial two-fold dilution of the test substance in agar that is then inoculated with the bacterial organism, so that a range of concentrations are tested. The minimum concentration that can create inhibition is regarded as the MIC. Hence, to calculate the ΣFIC, several MIC values are required, then the calculations follow Eqs. 1–3.

In Eq. 1 the concentration of A (Con.A) in the mixture of drugs A+ B at the combined MIC concentration, is divided by the MIC of drug A alone (MIC-A) to give FIC-α. In Eq. 2 the concentration of B (Con.B) in MIC A+ B is divided by the MIC of drug B alone to give FIC-β. In Eq. 3 FIC-α is combined with FIC-β (α+β) to give the ΣFIC value (Sueke et al., 2010). For example, if the MIC value of A + B is 0.5 mg ml^−1^ at a ratio of 1:4 of A:B, then Con. A is 0.1 and Con. B is 0.4 mg ml^−1^. If the MIC value of A alone is 1.5 mg ml^−1^ and B alone is 1.0 mg ml^−1^ then FIC-α is 0.1/1.5 = 0.07 and FIC-β is 0.4/1.0 = 0.4; then the ƩFIC value is 0.07 + 0.4 = 0.47.

$$FIC‐\alpha =\frac{Con.A\;\left(in\;MIC\;A+B\right)}{MIC‐A}$$ (1)

$$FIC‐\beta =\frac{Con.B\;\left(in\;MIC\;A+B\right)}{MIC‐B}$$ (2)

ΣFIC=FIC‐α+FIC‐β (3)

When the ΣFIC is ≤ 0.5 it is synergistic, if > 0.5–1.0 it is additive, if > 1.0 – ≤4.0 it is noninteractive and >4.0 makes it antagonistic (Van Vuuren and Viljoen, 2011).

The distinction between synergism and potentiation needs to be made. Synergism is defined by the increase of activity by a combination of two ‘active’ compounds. In synergism, enhancement of the activity is greater than the sum of the two effectors (A+ B) giving 1 + 1 ≠ 2 or 1 + 1 = >2. Alternatively, a subset of synergism is potentiation, which occurs when a non-active compound enhances the activity of the active compound (i.e., 1 + 0 = >1). The distinction is usually only made in the context of defeating resistance mechanisms, i.e., if one component blocks an efflux channel to augment the effect of an antibiotic, it is regarded as the potentiator (Mandeville and Cock, 2018).

While *in vivo* synergism can be caused by a wider range of system interactions, such as by changing a xenobiotic’s pharmacokinetics by slowing its metabolism, *in vitro* synergism has a narrower range, which is commonly the outcome of targeting two different mechanisms to achieve an enhanced outcome, including resistance mechanisms of pathogenic microbes. This means that by testing against a single target, such as a single enzyme, synergism is not possible, i.e., synergism requires at least a whole cell to manifest. Because most essential oil components confer effects to cell walls of bacteria and eukaryotes, their synergistic effects when combined with compounds that have specific targets, are caused by destabilising the walls of target cells.

In many synergism studies, essential oils and volatile organic compounds are regarded as non-active participants in combination with pharmaceuticals, so they are described as potentiators. While other researchers require stronger effects from antimicrobials, most researchers consider an MIC at <1 mg mL^−1^ as active (Van Vuuren and Holl, 2017), which is common in essential oils research. Consequently, the terms synergistic and potentiation are often used at the discretion of the authors in the published literature.

The most common potentiating effects described for volatile organic compounds or essential oils in the literature is focused on combinations with antibiotics from ‘big pharma’, i.e., essential oils from *Thymus vulgaris* L synergistically enhance the antibiotic cefixime (Jamali et al., 2017). In the pharmaceutical world the use of volatile organic compounds on their own to enact antimicrobial outcomes is not feasible for economic reasons. The concentrations must be many orders of magnitude higher to be comparable to microbially derived antibiotics (Sadgrove and Jones, 2019), which raises the cost of production to beyond reasonable, and limits the range of applications to topical use only (inhalation, topical dermal or gastro/intestinal epithelial). Hence, rather than being antimicrobial *per se*, volatile organic compounds are appropriately thought of as antiseptic compounds (Kon and Rai, 2012), with only general specificity in the mechanism of action. However, synergistic or potentiation effects are still of interest to pharma, by antagonising resistance mechanisms in pathogenic strains. The most commonly cited potentiation effect ascribed to plant metabolites is the attenuated effects of efflux ‘pumps’ (Khameneh et al., 2019). Prokaryotic efflux pumps are bacterial or viral membrane bound channels called ‘transport proteins’ that promote the disposal of cellular waste or toxins. Gene modulation effects by volatile organic compounds also occur in the prokaryotic cells of pathogenic microbes, which involves the downregulation of resistance associated genes (Chovanová et al., 2016), leading to the potentiation of other antimicrobial metabolites or antibiotics. Furthermore, volatile organic compounds have also shown the ability to downregulate expression of genes responsible for pathogen toxin secretion (Khoury et al., 2016), which attenuates virulence.

Normally the excretion of antimicrobial drugs via efflux pumps does not antagonise drug efficacy, but with the new trends involving overexpression of multidrug resistance efflux pump genes (Blanco et al., 2016), antibiotics are becoming less efficacious. Inhibiting this mechanism causes the accumulation of the antimicrobial drug in the bacteria’s cytoplasm, which enables an active concentration of the drug to be reached (Bambeke et al., 2003). While there are no efflux pump inhibitors in wider clinical use, volatile organic compounds are known to have this effect (Mikulášová et al., 2016). For example, the sesquiterpenes *epi*-cubenol and 15-copaenol were able to produce ΣFIC values in the range of 0.03–0.26 in combination with standard antibiotics against strains of *Staphylococcus aureus* that overexpress the NorA gene for the NorA efflux pump. These potentiating effects are attributed to efflux pump inhibition at a concentration of sesquiterpene that is less than 0.25 μg ml^−1^ (Espinoza et al., 2019), a concentration that is feasible in blood plasma alone, discounting accumulating effects in the body’s tissues.

Essential oil components may also augment the efficacy of other drugs by enhancement of their penetration (Aqil et al., 2007; Chen et al., 2015). The mechanism is thought to be related to disruption of the highly ordered structure of the stratus corneum lipid, leading to an increase in the intercellular diffusivity. This is established by the observation of a shift from ‘*trans* to *gauche*’ conformation in the methylene carbons along the alkyl chain of lipids (Chen et al., 2015). In the case of paracetamol, penetration enhancement values for each of the essential oils correlate to the skin permeation profiles of the individual essential oils. Hence, penetration enhancement can be predicted from the flux (μg.cm^−2^. h^−1^) or Q48 (μg.cm^−2^) of the individual essential oil. Essential oils with pronounced permeation values were produced by clove, angelica and chuanxiong and the most represented components were ligustilide and eugenol (Chen et al., 2015), which have the character of delocalised electrons (aromatic rings).

Furthermore, the cell and organelle wall permeabilising effects of lipophilic compounds can potentiate the influx of other exogenous compounds that confer gene modulatory effects. Although not a great deal of research has focused on this, the whole range of compounds in extracts from aromatic species may have synergistic or potentiation effects. Extracts from aromatic species have a range of compounds in their extracts, which include fixed (non-volatile) and volatile metabolites. As an example, the aromatic species *Elytropappus rhinocerotis* (L.f) Less. is used in South Africa as a treatment against foot odours and infections. The extract includes volatile organic compounds and non-volatile labdane diterpenes, both of which antagonised the growth of food fungal pathogens and odour causing bacteria (i.e., *Brevibacterium agri*) (Hulley et al., 2019).

The possibility for synergism between essential oil components and cannabinoids has been explored in theory (Russo, 2011). However, limited research has been conducted to specifically address these questions. The dominant sesquiterpene β-caryophyllene has been the focus of many studies, including as a synergist in antimicrobial outcomes. A recent study of *Vepris gossweileri* I. Verd., discovered several antimicrobial synergisms with β-caryophyllene in an extract of the leaves, which was defined as a ‘multi-layered’ synergism (Langat et al., 2021). Synergism against a model yeast and Gram-positive organism was demonstrated between β-caryophyllene and minor alkaloids with a ΣFIC value of 0.02. However, the synergistic effects were augmented by the chlorophyll derivative pheophytin A. In a follow-up study (results unpublished) it was realized that chlorophyll, pheophytins or pheophorbides are potent antimicrobial synergists in combination with β-caryophyllene. Hence, antimicrobial effects of green plant extracts, such as cannabis or CBD oil, are likely to be the result of a synergism between β-caryophyllene and the chlorophyll derivatives.

Another study of synergism argued that essential oil components in roots of *Citrus* x *limon* (L.) Osbeck synergised with specific methoxylated flavonoids released out of decomposing leaf litter at the base of the tree, providing protective effects against pathogenic root fungi (Nsangou et al., 2021). A hypothetical that is inspired by this research outcome involves antimicrobial synergisms against ruminant organisms that reside in the gut of herbivorous insects. This has not yet been examined in detail but stands out as a high possibility. Otherwise, antimicrobial synergisms may be considered as an important part of plant defence and the wider scientific community is encouraged to examine this in more detail, because there are both ecological and health-related implications from such research, i.e., there are many ways that such synergisms can be utilised in human health as prebiotics for the gastrointestinal tract.

### Mammalian Gene Regulation and Immunomodulation

While it is necessary for volatile organic compounds to reach high concentrations to confer ‘contact’ antimicrobial effects, gene-modulation can occur at concentrations that are many folds’ lower. Mammalian cells have diverse super-families of transcription factors that have lipid binding domains that inevitably become the target of lipophilic compounds. One study that focused on the dermal fibroblast demonstrated upregulation of hundreds of genes associated with (anti)inflammation, metabolism and proliferation, which occurs at a concentration of less than 100 μg g^−1^ (0.01%) by a variety of volatile compounds (Han and Parker, 2017). The genes are modulated uniquely by each of the compounds tested, at concentrations that are low enough to be feasible in topical applications or tissue bioaccumulation, without cytotoxic effects (2017).

In the context of human (eukaryotic) cells, modulation of the expression of peroxisome proliferator-activated receptor (PPAR) genes can occur in response to essential oil components (Goto et al., 2010). For example, limonene at 10 μM (1.5 μg g^−1^) increased phosphorylation of Akt leading to enhanced glucose uptake in adipocytes, promoted adipocyte differentiation and also allegedly the expression of PPAR-γ genes (Soundharrajan et al., 2018). Linalool appears to do the opposite, by inhibiting adipocyte differentiation (Cheng et al., 2018), which may be useful in obesity control. Nevertheless, both compounds can reach systemic concentrations of 2 μg ml^−1^ in humans, by topical and oral application, with no adverse effects.

PPAR agonism is also associated with anti-inflammatory effects (immunomodulation) by inhibition of interleukin-1 induced cyclooxygenase-2 expression (Hotta et al., 2010). However, immunomodulation by essential oil components is not limited to this effect. The literature describes the modifying effects on secretion of a wide diversity of cytokines other than interleukin-1 (Valdivieso-Ugarte et al., 2019), which can occur by agonism of nuclear or membrane receptors, such as the cannabinoid receptors, but the details are often not explained. For example, systemic treatment using 50 mg kg^−1^ essential oil from *Cordia verbenacea* A. DC reduced tumour necrosis factor (TNF-α) production, which interrupted the inflammatory cascade induced by carrageenan (Passos et al., 2007). Ingestion of 50 mg kg^−1^ of the two main volatile organic compounds, α-humulene and *E*-caryophyllene, also reduced inflammation. *E*-Caryophyllene only diminished TNF-α release whereas α-humulene also interrupts interleukin-1β, cyclooxygenase-2, nitric oxide and prostaglandin E-2 (PGE(2)) (Fernandes et al., 2007). Furthermore, inflammation was greatly attenuated by oral treatment an hour before lipopolysaccharide (LPS) was used as an inducer, evidently by the same mechanism as above (Medeiros et al., 2007). In topical applications a much lower concentration is required. Hence, a commercial product named Acheflan with *C. verbenacea* essential oil as an active ingredient is available in Brazil as a topical anti-inflammatory. Pharmacokinetic studies of the main sesquiterpene, α-humulene, using oral and intravenous doses of 1,000 mg kg^−1^ mouse, demonstrated that peak plasma concentrations can reach from 2–20 μg ml^−1^ without adverse effects in the short term (Chaves et al., 2008).

Before the term ‘potentiator’ came into practice, researchers used the former term ‘entourage effect’ to describe the potentiating effects of volatile organic compounds from the marijuana variety of *Cannabis sativa*. The psychoactive effects from marijuana are caused by tetrahydrocannabinol, which is a potent agonist for cannabinoid receptor-1. However, as previously mentioned, the headspace of marijuana also includes the volatile sesquiterpene β-caryophyllene, which is regarded as a phytocannabinoid that is not psychoactive because it is a selective agonist of cannabinoid receptor-2 (CB2), a receptor in immune cells (Gonçalves et al., 2020). Caryophyllene is the most publicised example of a specific CB2 agonist (Francomano et al., 2019). It is known to promote wound healing in dermal skin models by following multiple routes, but the anti-inflammatory effects are likely to be the most important in this outcome. Concomitant with higher rates of re-epithelialization is the upregulated expression of hair follicle bulge stem cells, which has strong implications to hair health (Koyama et al., 2019).

But the effects of β-caryophyllene are not restricted to cannabinoid receptors. β-Caryophyllene positively regulates the pI3K/Akt/mTOR signalling pathway in tissues that express Akt3, a protein kinase B isoform important for the regulation of neuronal development. Alternatively, in liver cells and T lymphocytes this pathway is negatively regulated by the same treatment but upregulated in neuronal cells, indicating a role in tissue-specific inflammation treatment. Regulation of the pI3K/Akt/mTOR pathway is entirely dependent on Akt3, meaning that it makes sense that the JAK/STAT signalling pathway is upregulated independently. Hence, the essential oil of copaiba that is rich in β-caryophyllene confers gene regulatory effects that differ according to the tissue (Urasaki et al., 2020), i.e., copaiba essential oil can confer anti-inflammatory effects without dulling the immune response. Furthermore, it was demonstrated that by upregulation of the pI3k/Akt/mTOR pathways in the dermis, promotion of reepithelization of superficial wounds occurs (Koyama et al., 2019).

In contrast, the gingerols from *Zingiber officinale* allegedly downregulate the pI3k/Akt/mTOR pathway (Wang et al., 2016). The gingerols are also potent antioxidants (Mao et al., 2019). Together these effects confer protection against oxidative species generation from mitochondrial respiration. Previously it was thought that anecdotal accounts of use of ginger for hair restoration, in men living with androgenetic alopecia, were a contradiction because it slowed the growth of dermal papilla cells *in vitro* (Miao et al., 2013), however evidence now indicates the Akt-mTOR pathway is overactive in bald scalps and this process is associated with overproduction of reactive oxygen species (Sadgrove, 2021).

A bit like CBD oil, the anti-inflammatory effects of essential oil components may also play a role in aiding sleep. A famous sleep-inducing herbal tea is chamomile (*Matricaria recutita* L., Asteraceae). The blue colour of the essential oil is caused by chamazulene, which is an anti-inflammatory component that is active at 10–60 μg ml^−1^ *in vitro* (Ma et al., 2020). However, chamazulene is a derivative produced in hydrodistillation by conversion of the precursor matricine, which is the version that is present in chamomile tea. Matricine is active at a lower concentration, inhibiting NF-KB activation within the margin of 3–22 μg ml^−1^ (Flemming et al., 2015). Because NF-KB activation is associated with sleep deprivation (Irwin and Wang, 2008), the link between chamomile tea and restful sleep may be in the anti-inflammatory effects of matricine, or its combined effects with the non-volatile flavonoid component quercetin (Kambe et al., 2010). Alternatively, mice treated with essential oil from chamomile were observed to have lower plasma histamine levels than control after challenging with 2,4-dinitrochlorobenzene (Lee et al., 2010), which conveys that the essential oil may be an antihistamine and antihistamines are known to induce sleep or drowsiness.

Other essential oil components that have been demonstrated as anti-inflammatory by *in vitro* assay of lipopolysaccharide induced cytokine release include the santalol isomers from sandalwood (Sharma et al., 2014), eugenol from clove (Saad et al., 2013), and carvacrol from thyme (Hotta et al., 2010) among others. The numbers of essential oil components associated with anti-inflammatory effects are numerous and mechanisms are often not explained but it is reasonable to hypothesise a role for PPARS.

### Areas for Further Research

#### Cardiovascular Disease

Essential oil components are worthy of further consideration in the context of cardiovascular disease prophylaxis. It is the contention of this narrative that aromatic foods that are included in the diet in the long term enact positive effects that interrupt the aetiological progression of many forms of disease, particularly cardiovascular diseases. However, prophylactic effects are only realised over the course of decades, so it is difficult to prove *in vivo*. Nevertheless, there is mounting indirect evidence to support this hypothesis. For example, dietary *d* -limonene has demonstrated insulin sensitising effects and reduced oxidative stress in rats fed on an obesogenic diet (Santiago et al., 2012). Because insulin resistance is regarded as a risk factor (Petrie et al., 2018), then it is feasible that attenuation of negative effects associated with insulin resistance is prophylactic for cardiovascular disease.

There are many essential oil components that confer insulin sensitising effects alone (Sebai et al., 2013; Hasanein and Riahi, 2015) or as part of a combination of fatty oils and plant extracts (Talpur et al., 2004). Authors of these types of studies offer the explanation that essential oil components help the body’s cells to cope with oxidative stress, either by direct radical quenching or modulation of antioxidant genes (Liu et al., 2013; Mohamed et al., 2016), and further to confer anti-inflammatory effects, all of which attenuate insulin resistance.

According to the modern paradigm of cardiovascular disease, chronic inflammation is considered as the root of its pathogenesis. One group of authors argue that the comorbidities of cardiovascular disease are characterised by chronic systemic inflammation and propose that if untreated will lead to heart disease (Bigeh et al., 2020). Chronic systemic inflammation has two main dietary triggers, with the first being obesogenic eating (de Luca and Olefsky, 2008), leading into high caloric loading and reactive oxygen species generation, mitochondrial burnout and activation of the polyol pathway (Johnson et al., 2017).

Considering the strong link between inflammation and the eventual development of cardiovascular diseases, dietary inclusion of anti-inflammatory phytochemicals over a long period of time may be considered prophylactic. However, it must be considered if volatile organic compounds can be raised to high enough concentrations in plasma to achieve the anti-inflammatory effects demonstrated *in vitro*. Fortunately, it has already, been demonstrated in rats that many of the anti-inflammatory essential oil components are feasibly raised to the required plasma concentrations by dietary application at quantities present in a serving of aromatic food, but the mechanism as explained by *in vitro* studies are not necessarily the actual mechanisms *in vivo*. For example, *in vitro* inflammation in macrophages stimulated by TNF-α and nitric oxide was attenuated by the essential oil components of *Cinnamomum zeylanicum* Blume at concentrations of 7.5–8.6 μg ml^−1^ for *E*-cinnamaldehyde or 5.7–12.6 μg ml^−1^ for O-methoxycinnamaldehyde (Gunawardena et al., 2015). With consideration to the cytochrome P450 inhibiting effects of *E*-cinnamaldehyde (Chan et al., 2016), these concentrations may be more easily met in blood plasma than other types of monoterpenes, however it is unclear if these plasma concentrations can be feasibly met in humans (Zhu et al., 2017), or if the metabolic products cinnamic acid, cinnamyl alcohol or methyl cinnamate also enact anti-inflammatory effects. Nevertheless, *in vivo* effects are achievable in male Wistar rats at an oral dose of 143.8 μmol kg^−1^ daily (Farrokhfall et al., 2010). Generally *in vivo* studies that demonstrate positive outcomes followed a repeated dosing regime, rather than a single oral dose. Hence, the effects may be related to accumulation of essential oil components and their respective metabolites in tissues and changes to the expression of metabolising enzymes in liver and the dermis.

As mentioned earlier, the mechanism of anti-inflammatory effects of essential oil components may be enacted by agonism of peroxisome proliferator activated receptors (PPAR) (Goto et al., 2010; Hotta et al., 2010; Katsukawa et al., 2010; Li et al., 2015), because PPARS are important modulators of inflammation (Daynes and Jones, 2002). The concentrations required to achieve agonism of PPARS are similar to the concentrations in studies describing anti-inflammation in macrophages, i.e., cinnamaldehyde activated PPARS at 1.3–6.6 μg ml^−1^ (Li et al., 2015). However, because the PPARS are concentrated in adipose tissues and liver, then the concentrations of xenobiotic essential oil components will be many folds higher in the vicinity of PPARS. Hence, these effects are feasible *in vivo* with moderate consumption of aromatic foods, i.e., rats fed *d* -limonene demonstrated significant upregulation of PPARα genes (Jing et al., 2013). Because PPARS are also important in the action of insulin signalling and blood glucose control (Leonardini et al., 2009) this may also explain the mechanism of diabetic control by oral essential oil in rat studies.

The second leading cause of systemic inflammation is gastrointestinal bacterial dysbiosis (Jin et al., 2018). The problem starts with ‘leaky gut’, which results from intestinal inflammation as a response to bacterial overgrowth. Due to damage to the mucosal or epithelial barrier bacterial lipopolysaccharides enter into the lining and cross in portal circulation (Onal et al., 2019). In cases of more severe disturbance to the intestinal epithelial barrier function, live bacteria escape the gut lumen and translocate into systemic circulation, contributing to atherosclerotic symptoms and myocardial infarction (Zhou et al., 2018). The key to attenuating this problem lies in strengthening the intestinal epithelial barrier via the nurturing of commensal gut bacteria and attenuation of bacterial overgrowth (Ohland and Macnoughton, 2010).

Hence, the use of aromatic plant foods as prebiotics may be considered prophylactic for cardiovascular disease. As previously mentioned, synergisms between essential oil components and chlorophyll or the derivatives, pheophytin or pheophorbide, is a worthy research undertaking. The possibility of controlling bacterial overgrowth in the intestinal space is a neglected but important vision in the prebiotic initiative (Zhong et al., 2017). In this regard, controlling bacterial overgrowth attenuates or prevents inflammation, enhance re-epithelialization, and closes the barrier between portal circulation and bacterial lipopolysaccharide.

### Safety and Chemoprevention With Volatile Organic Compounds

Because essential oil components accumulate in the body’s tissues, the obstacle of bioavailability may be overcome, particularly in cancers. As previously mentioned, metabolite conjugation reduces a compound’s bioavailability and prevents it from reaching a potentially toxic concentration in normal tissue, but in cancerous tissue deconjugation reverses the phase 2 metabolism and causes a localised build-up of preconjugated xenobiotics. The prooxidant effects (Burt, 2004) that are normally not occurring in healthy tissue are enabled by this localised concentration of xenobiotics, which include a host of ingested plant-derived secondary metabolite, including essential oil components.

Generally, phase 1 metabolism makes oxidised derivatives of essential oil components and in phase 2 metabolism they are conjugated to either a glucuronide, glutathione or a sulphate moiety (Sadgrove and Jones, 2019). Although this process is thought to make the respective xenobiotic completely unavailable, it is now known that deconjugation processes return xenobiotics to their active pre-conjugated forms. These effects are well known for non-volatile plant compounds, such as curcumin, which is rapidly metabolised into a glucuronide that is regarded as unavailable, but is transformed back into the aglycone by β-glucuronidase activity in bone tissue undergoing osteoclastogenesis (Kunihiro et al., 2019). Previously several essential oils were discovered as preventors of bone reabsorption (osteoclast breakdown) (Mühlbauer et al., 2003), and the modern realization of the β-glucuronidase activity clarifies how the essential oil components can meet the required concentrations. A similar scenario was observed with the non-volatile metabolite resveratrol, which is quickly metabolised into a sulphate conjugate that is reversed by steroid sulphatases in cancer cells, inducing autophagy (Andreadi et al., 2014).

Cancers express high levels of β-glucuronidase (Su et al., 2014) and steroid sulphatases (Foster, 2021). Hence, conjugated xenobiotics may be regarded as glucuronide or sulphate prodrugs that are activated on-site to enact therapeutic effects (de Graaf et al., 2002). Although this type of research has not focused on essential oil components, the anticancer effects of phase 1 metabolites are sometimes examined by forward thinking researchers, which are the likely forms that appear in tumours or cancers when deconjugation occurs. For example, *d* -limonene was previously considered a worthy candidate in chemotherapy, and the phase 1 metabolites were also of interest in this context (da Silveira e Sá et al., 2013).

Research on the anticancer effects of essential oil components generally focuses on cytotoxic effects caused by various mechanisms, including overexpression of liver detoxification enzymes, changes to the membrane potential of mitochondria (causing leakage of free radicals) and whole cancer cells, prooxidant effects (mainly by arenes), inhibition of angiogenesis, and modification of tumour-inducing genes (Bhalla et al., 2013; Legards et al., 2014). Complementary, additive or synergistic effects, where a supportive role to the more conventional treatment, is also a point of interest in modern research (Legards et al., 2014). However, the insulin sensitising effect of essential oil components (Talpur et al., 2004) is a worthy consideration, because modulation of glucose metabolism in cancers has also become a point of interest in modern research (Hay, 2016).

Alternatively, lifestyles that include diets fortified with aromatic species may be preventative or antagonistic of oncogenesis. This school of thought is known widely as a chemoprevention strategy. Hence, the powers of plant-derived xenobiotics, such as essential oil components, are more than likely realized as preventative because of the anticancer subtly by comparison with conventional chemotherapy drugs. Milder nature-based chemotherapy metabolites, such as essential oil components, can be endured by the human body over the long term, meaning that cancerous cells can be antagonised before they establish as larger resilient cysts. Furthermore, anticancer research of essential oils should focus on the possible antagonism of cancer metastasis during the remission period between conventional anticancer treatments. This is a feasible initiative because it requires following cancer survivors and quantifying those who stay in remission whilst incorporating a plant-based health regime.

## References

[B1] Abd El-Moneim M. R. Fatma S. A. Turky A. F. (2012). Control of Tetranychus Urticae Koch by Extracts of Three Essential Oils of Chamomile, Marjoram and Eucalyptus. Asian Pac. J. Trop. Biomed. 2, 24–30. 10.1016/S2221-1691(11)60184-6 23569829 PMC3609209

[B2] Adams R. P. (2007). Identification of Essential Oil Components by Gas Chromatography/mass Spectrometry. Carol Stream, IL: Allured Publishing Corporation. ISBN 978-1-932633-21-4.

[B3] Amiresmaeili A. Roohollahi S. Mostafavi A. Askari N. (2018). Effects of Oregano Essential Oil on Brain TLR4 and TLR2 Gene Expression and Depressive-like Behavior in a Rat Model. Res. Pharm. Sci. 13, 130–141. 10.4103/1735-5362.223795 29606967 PMC5842484

[B4] Anastasiou C. Buchbauer G. (2017). Essential Oils as Immunomodulators: Some Examples. Open Chem. 15, 352–370. 10.1515/chem-2017-0037

[B5] Andreadi C. Britton R. G. Patel K. R. Brown K. (2014). Resveratrol-sulfates Provide an Intracellular Reservoir for Generation of Parent Resveratrol, Which Induces Autophagy in Cancer Cells. Autophagy 10, 524–525. 10.4161/auto.27593 24419144 PMC4077892

[B6] Aqil M. Ahad A. Sultana Y. Ali A. (2007). Status of Terpenes as Skin Penetration Enhancers. Drug Discov. Today 12, 1061–1067. 10.1016/j.drudis.2007.09.001 18061886

[B7] Asakawa Y. Ludwiczuk A. (2013). “Bryophytes: Liverworts, Mosses, and Hornworts: Extraction and Isolation Procedures,” in Metabolomics Tools for Natural Product Discovery. Editors Roessner U. Dias D. A. (Totowa, NJ: Humana Press), 1–20. 10.1007/978-1-62703-577-4_1 23963899

[B8] Ashour M. L. Youssef F. S. Gad H. A. Wink M. (2017). Inhibition of Cytochrome P450 (CYP3A4) Activity by Extracts from 57 Plants Used in Traditional Chinese Medicine (TCM). Pharmacogn Mag. 13, 300–308. 10.4103/0973-1296.204561 28539725 PMC5421430

[B9] Bakkali F. Averbeck S. Averbeck D. Idaomar M. (2008). Biological Effects of Essential Oils-Aa Review. Food Chem. Toxicol. 46, 446–475. 10.1016/j.fct.2007.09.106 17996351

[B10] Banerjee S. Sharma R. Kale R. K. Rao A. R. (1994). Influence of Certain Essential Oils on Carcinogen-Metabolizing Enzymes and Acid-Soluble Sulfhydryls in Mouse Liver. Nutr. Cancer 21, 263–269. 10.1080/01635589409514324 8072879

[B11] Bauer K. Garbe D. (1985). Common Fragrance and Flavor Materials. Preparation, Properties and Uses. Weinheim: VCH Verlagsgesellschaft.

[B12] Bedini S. Farina P. Napoli E. Flamini G. Ascrizzi R. Verzera A. (2021). Bioactivity of Different Chemotypes of Oregano Essential Oil against the Blowfly *Calliphora Vomitoria* Vector of Foodborne Pathogens. Insects 12, 52. 10.3390/insects12010052 33440619 PMC7826667

[B13] Beyer J. Ehlers D. Maurer H. H. (2006). Abuse of Nutmeg (Myristica Fragrans Houtt.): Studies on the Metabolism and the Toxicologic Detection of its Ingredients Elemicin, Myristicin, and Safrole in Rat and Human Urine Using Gas Chromatography/mass Spectrometry. Ther. Drug Monit. 28, 568–575. 10.1097/00007691-200608000-00013 16885726

[B14] Bhalla Y. Gupta V. K. Jaitak V. (2013). Anticancer Activity of Essential Oils: a Review. J. Sci. Food Agric. 93, 3643–3653. 10.1002/jsfa.6267 23765679

[B15] Bigeh A. Sanchez A. Maestas C. Gulati M. (2020). Inflammatory Bowel Disease and the Risk for Cardiovascular Disease: Does All Inflammation lead to Heart Disease? Trends Cardiovasc. Med. 30, 463–469. 10.1016/j.tcm.2019.10.001 31653485

[B16] Blanco P. Hernando-Amado S. Reales-Calderon J. A. Corona F. Lira F. Alcalde-Rico M. (2016). Bacterial Multidrug Efflux Pumps: Much More Than Antibiotic Resistance Determinants. Microorganisms 4, 14. 10.3390/microorganisms4010014 PMC5029519 27681908

[B17] Bowles J. E. (2003). The Chemistry of Aromatherapeutic Oils. Crows Nest, NSW Australia: Allen & Unwin.

[B45] Brain L. J. Green D. M. Api A. M. (2007). *In Vitro* Human Skin Penetration of the Fragrance Material Linalool. Toxicologist 102 (1), 319. 10.1016/j.tiv.2006.08.005 17045775

[B18] Buchbauer G. (2010). “Chapter 9. Biological Activities of Essential Oils,” in Handbook of Essential Oils: Science, Technology and Applications. Editors Başer K. H. C. Buchbauer G. (London: CRC Press, Taylor and Francis Group).

[B19] Burt S. (2004). Essential Oils: Their Antibacterial Properties and Potential Applications in Foods-Aa Review. Int. J. Food Microbiol. 94, 223–253. 10.1016/j.ijfoodmicro.2004.03.022 15246235

[B20] Cal K. (2006). Skin Penetration of Terpenes from Essential Oils and Topical Vehicles. Planta Med. 72, 311–316. 10.1055/s-2005-916230 16557471

[B21] Chan J. Oshiro T. Thomas S. Higa A. Black S. Todorovic A. (2016). Inactivation of CYP2A6 by the Dietary Phenylpropanoid Trans-cinnamic Aldehyde (Cinnamaldehyde) and Estimation of Interactions with Nicotine and Letrozole. Drug Metab. Dispos 44, 534–543. 10.1124/dmd.115.067942 26851241 PMC4810772

[B22] Chaves J. S. Leal P. C. Pianowisky L. Calixto J. B. (2008). Pharmacokinetics and Tissue Distribution of the Sesquiterpene Alpha-Humulene in Mice. Planta Med. 74, 1678–1683. 10.1055/s-0028-1088307 18951339

[B23] Chen J. Jiang Q. D. Chai Y. P. Zhang H. Peng P. Yang X. X. (2016). Natural Terpenes as Penetration Enhancers for Transdermal Drug Delivery. Molecules 21, 1709. 10.3390/molecules21121709 PMC6273457 27973428

[B24] Chen J. Jiang Q. D. Wu Y. M. Liu P. Yao J. H. Lu Q. (2015). Potential of Essential Oils as Penetration Enhancers for Transdermal Administration of Ibuprofen to Treat Dysmenorrhoea. Molecules 20, 18219–18236. 10.3390/molecules201018219 26457698 PMC6332003

[B25] Cheng B. H. Sheen L. Y. Chang S. T. (2018). Hypolipidemic Effects of S-(+)-linalool and Essential Oil from *Cinnamomum Osmophloeum* Ct. Linalool Leaves in Mice. J. Tradit Complement. Med. 8, 46–52. 10.1016/j.jtcme.2017.02.002 29321988 PMC5755959

[B26] Choi J. Lee K. T. Ka H. Jung W. T. Jung H. J. Park H. J. (2001). Constituents of the Essential Oil of the Cinnamomum cassia Stem Bark and the Biological Properties. Arch. Pharm. Res. 24, 418–423. 10.1007/BF02975187 11693543

[B27] Chovanová R. Mikulášová M. Vaverková Š. (2016). Modulation of mecA Gene Expression by Essential Oil from Salvia Sclarea and Synergism with Oxacillin in Methicillin Resistant Staphylococcus Epidermidis Carrying Different Types of Staphylococcal Chromosomal Cassette Mec. Int. J. Microbiol. 2016, 6475837. 10.1155/2016/6475837 26880926 PMC4736799

[B28] Clevenger J. F. (1928). Apparatus for the Determination of Volatile Oil*. J. Am. Pharm. Assoc. (1912) 17, 345–349. 10.1002/jps.3080170407

[B29] Collins T. L. Andrew R. L. Greatrex B. W. Bruhl J. J. (2018). Reliable Analysis of Volatile Compounds from Small Samples of *Eucalyptus Magnificata* (Myrtaceae). Aust. Syst. Bot. 31, 232–240. 10.1071/sb18005

[B160] Damjanovic B. Skala D. Baras J. Petrovic-Djakov D. (2006). Isolation of essential oil and supercritical carbon dioxide extract of Juniperus communis L. fruits from Montenegro. Flavour Fragr. J. 21, 875–880.

[B30] Dawidowics A. L. Dybowski M. P. (2014). Simple SPE–GC Method for Anethole Determination in Human Serum. J. Sep. Sci. 37, 393–397. 24302657 10.1002/jssc.201300999

[B31] Daynes R. A. Jones D. C. (2002). Emerging Roles of PPARS in Inflammation and Immunity. Nat. Rev. Immunol. 2, 748–759. 10.1038/nri912 12360213

[B32] de Cássia da Silveira e Sá R. Andrade L. De Sousa D. (2013). A Review on Anti-inflammatory Activity of Monoterpenes. Molecules 18, 1227–1254. 10.3390/molecules18011227 23334570 PMC6269770

[B33] De Graaf M. Boven E. Scheeren H. W. Haisma H. J. Pinedo H. M. (2002). Beta-Glucuronidase-Mediated Drug Release. Curr. Pharm. Des. 8, 1391–1403. 10.2174/1381612023394485 12052215

[B34] De Luca C. Olefsky J. M. (2008). Inflammation and Insulin Resistance. FEBS Lett. 582, 97–105. 10.1016/j.febslet.2007.11.057 18053812 PMC2246086

[B35] Dobreva A. Kovatcheva N. Astatkie T. Zheljazkov V. D. (2011). Improvement of Essential Oil Yield of Oil-Bearing (*Rosa Damascena* Mill.) Due to Surfactant and Maceration. Ind. Crops Prod. 34, 1649–1651. 10.1016/j.indcrop.2011.04.017

[B36] El Khoury R. Atoui A. Verheecke C. Maroun R. El Khoury A. Mathieu F. (2016). Essential Oils Modulate Gene Expression and Ochratoxin A Production in *Aspergillus carbonarius* . Toxins (Basel) 8, 242. 10.3390/toxins8080242 PMC4999858 27548221

[B37] Espinoza J. Urzúa A. Sanhueza L. Walter M. Fincheira P. Muñoz P. (2019). Essential Oil, Extracts, and Sesquiterpenes Obtained from the Heartwood of Pilgerodendron Uviferum Act as Potential Inhibitors of the *Staphylococcus aureus* NorA Multidrug Efflux Pump. Front. Microbiol. 10, 337. 10.3389/fmicb.2019.00337 30863385 PMC6400098

[B38] Farrokhfall K. Khoshbaten A. Zahediasl S. Mehrani H. Karbalaei N. (2010). Improved Islet Function Is Associated with Antiinflammatory, Antioxidant and Hypoglycemic Potential of Cinnamaldehyde on Metabolic Syndrome Induced by High Tail Fat in Rats. J. Funct. Foods 10, 397–406.

[B39] Fernandes E. S. Passos G. F. Medeiros R. Da Cunha F. M. Ferreira J. Campos M. M. (2007). Anti-inflammatory Effects of Compounds Alpha-Humulene and (-)-Trans-Caryophyllene Isolated from the Essential Oil of *Cordia Verbenacea* . Eur. J. Pharmacol. 569, 228–236. 10.1016/j.ejphar.2007.04.059 17559833

[B40] Flemming M. Kraus B. Rascle A. Jürgenliemk G. Fuchs S. Fürst R. (2015). Revisited Anti-inflammatory Activity of Matricine *In Vitro*: Comparison with Chamazulene. Fitoterapia 106, 122–128. 10.1016/j.fitote.2015.08.010 26304764

[B41] Foster P. A. (2021). Steroid Sulphatase and its Inhibitors: Past, Present, and Future. Molecules 26, 2852. 10.3390/molecules26102852 34064842 PMC8151039

[B42] Francomano F. Caruso A. Barbarossa A. Fazio A. La Torre C. Ceramella J. (2019). β-Caryophyllene: A Sesquiterpene with Countless Biological Properties. Appl. Sci. 9, 5420. 10.3390/app9245420

[B43] Gonçalves E. C. D. Baldasso G. M. Bicca M. A. Paes R. S. Capasso R. Dutra R. C. (2020). Terpenoids, Cannabimimetic Ligands, beyond the *Cannabis* Plant. Molecules 25, 1567. 10.3390/molecules25071567 PMC7181184 32235333

[B44] Goto T. Takahashi N. Hirai S. Kawada T. (2010). Various Terpenoids Derived from Herbal and Dietary Plants Function as PPAR Modulators and Regulate Carbohydrate and Lipid Metabolism 2010, 9. PPAR Res. 10.1155/2010/483958 PMC2896613 20613991

[B46] Griffin G. Wray E. J. Martin B. R. Abood M. E. (1999). Cannabinoid Agonists and Antagonists Discriminated by Receptor Binding in Rat Cerebellum. Br. J. Pharmacol. 128, 684–688. 10.1038/sj.bjp.0702806 10516649 PMC1571656

[B47] Guimarães A. C. Meireles L. M. Lemos M. F. Guimarães M. C. C. Endringer D. C. Fronza M. (2019). Antibacterial Activity of Terpenes and Terpenoids Present in Essential Oils. Molecules 24, 2471. 10.3390/molecules24132471 PMC6651100 31284397

[B48] Gunawardena D. Karunaweera N. Lee S. Van Der Kooy F. Harman D. G. Raju R. (2015). Anti-inflammatory Activity of Cinnamon (C. Zeylanicum and C. cassia) Extracts - Identification of E-Cinnamaldehyde and O-Methoxy Cinnamaldehyde as the Most Potent Bioactive Compounds. Food Funct. 6, 910–919. 10.1039/c4fo00680a 25629927

[B49] Han X. Parker T. L. (2017). Essential Oils Diversely Modulate Genome-wide Gene Expression in Human Dermal Fibroblasts. Cogent Med. 4, 1307591. 10.1080/2331205x.2017.1307591

[B50] Hasanein P. Riahi H. (2015). Antinociceptive and Antihyperglycemic Effects of *Melissa Officinalis* Essential Oil in an Experimental Model of Diabetes. Med. Princ Pract. 24, 47–52. 10.1159/000368755 25402675 PMC5588194

[B51] Hay N. (2016). Reprogramming Glucose Metabolism in Cancer: Can it Be Exploited for Cancer Therapy? Nat. Rev. Cancer 16, 635–649. 10.1038/nrc.2016.77 27634447 PMC5516800

[B52] Hongratanaworakit T. Heuberger E. Buchbauer G. (2004). Evaluation of the Effects of East Indian Sandalwood Oil and Alpha-Santalol on Humans after Transdermal Absorption. Planta Med. 70, 3–7. 10.1055/s-2004-815446 14765284

[B53] Hotta M. Nakata R. Katsukawa M. Hori K. Takahashi S. Inoue H. (2010). Carvacrol, a Component of Thyme Oil, Activates PPARalpha and Gamma and Suppresses COX-2 Expression. J. Lipid Res. 51, 132–139. 10.1194/jlr.M900255-JLR200 19578162 PMC2789773

[B158] Houdkova M. Chaure A. Doskocil I. Havlik J. Kokoska L. (2021). Broth Macrodilution Volatilization Method for Antibacterial Susceptibility Testing of Volatile Agents and Evaluation of Their Toxicity Using Modified MTT Assay *In Vitro* . Molecules 26. 10.3390/molecules26144179 PMC8305236 34299454

[B159] Houdkova M. Rondevaldova J. Doskocil I. Havlik J. Kokoska L. (2017). Evaluation of Antibacterial Potential and Toxicity of Plant Volatile Compounds Using New Broth Microdilution Volatilization Method and Modified MTT Assay. Fitoterapia 118, 56–62. 28223069 10.1016/j.fitote.2017.02.008

[B54] Hulley I. M. Van Vuuren S. F. Sadgrove N. J. Van Wyk B. E. (2019). Antimicrobial Activity of *Elytropappus Rhinocerotis* (Asteraceae) against Micro-organisms Associated with Foot Odour and Skin Ailments. J. Ethnopharmacol 228, 92–98. 10.1016/j.jep.2018.09.014 30217789

[B55] Hulley I. M. Sadgrove N. J. Tilney P. M. Özek G. Yur S. Özek T. (2018). Essential Oil Composition of *Pentzia Incana* (Asteraceae), an Important Natural Pasture Plant in the Karoo Region of South Africa. Afr. J. Range Forage Sci. 35, 137–145. 10.2989/10220119.2018.1495265

[B56] Irwin M. R. Wang M. Ribeiro D. Cho H. J. Olmstead R. Breen E. C. (2008). Sleep Loss Activates Cellular Inflammatory Signaling. Biol. Psychiatry 64, 538–540. 10.1016/j.biopsych.2008.05.004 18561896 PMC2547406

[B57] Iso (2015). International Standards Organisation - Home Page. [Online]. Available: http://www.iso.org/iso/home.htm (Accessed, , 2015).

[B58] Jäger W. R. Buchbauer G. Jirovetz L. Fritzer M. (1992). Percutaneous Absorption of Lavender Oil from a Massage Oil. J. Soc. Cosmet. Chemists 43, 49–54.

[B59] Jamali C. A. Kasrati A. Fadli M. Hassani L. Leach D. N. Abbad A. (2017). Synergistic Effects of Three Moroccan Thyme Essential Oils with Antibiotic Cefixime. Phytothérapie, 1–6. 10.1007/s10298-017-1107-2

[B60] Jin M. Qian Z. Yin J. Xu W. Zhou X. (2018). The Role of Intestinal Microbiota in Cardiovascular Disease. J. Cel Mol Med 23, 2343–2350. 10.1111/jcmm.14195 PMC6433673 30712327

[B61] Jing L. Zhang Y. Fan S. Gu M. Guan Y. Lu X. (2013). Preventive and Ameliorating Effects of Citrus D-Limonene on Dyslipidemia and Hyperglycemia in Mice with High-Fat Diet-Induced Obesity. Eur. J. Pharmacol. 715, 46–55. 10.1016/j.ejphar.2013.06.022 23838456

[B62] Johnson R. J. Sánchez-Lozada L. G. Andrews P. Lanaspa M. A. (2017). Perspective: A Historical and Scientific Perspective of Sugar and its Relation with Obesity and Diabetes. Adv. Nutr. 8, 412–422. 10.3945/an.116.014654 28507007 PMC5421126

[B63] Jones G. L. Sadgrove N. J. (2015). “Ethnopharmacology in Australia and Oceania,” in Ethnopharmacology - A Reader. Editors Heinrich M. Jäger A. K. (West Sussex, UK: John Wiley & Sons).

[B64] Kambe D. Kotani M. Yoshimoto M. Kaku S. Chaki S. Honda K. (2010). Effects of Quercetin on the Sleep-Wake Cycle in Rats: Involvement of Gamma-Aminobutyric Acid Receptor Type A in Regulation of Rapid Eye Movement Sleep. Brain Res. 1330, 83–88. 10.1016/j.brainres.2010.03.033 20303338

[B65] Katsukawa M. Nakata R. Takizawa Y. Hori K. Takahashi S. Inoue H. (2010). Citral, a Component of Lemongrass Oil, Activates PPARα and γ and Suppresses COX-2 Expression. Biochim. Biophys. Acta 1801, 1214–1220. 10.1016/j.bbalip.2010.07.004 20656057

[B66] Khameneh B. Iranshahy M. Soheili V. Fazly Bazzaz B. S. (2019). Review on Plant Antimicrobials: a Mechanistic Viewpoint. Antimicrob. Resist. Infect. Control. 8, 118. 10.1186/s13756-019-0559-6 31346459 PMC6636059

[B67] Khumalo G. P. Sadgrove N. J. Van Vuuren S. Van Wyk B.-E. (2019). Antimicrobial Activity of Volatile and Non-volatile Isolated Compounds and Extracts from the Bark and Leaves of *Warburgia Salutaris* (Canellaceae) against Skin and Respiratory Pathogens. South Afr. J. Bot. 122, 547–550. 10.1016/j.sajb.2018.10.018

[B68] Kohlert C. Schindler G. März R. W. Abel G. Brinkhaus B. Derendorf H. (2002). Systemic Availability and Pharmacokinetics of Thymol in Humans. J. Clin. Pharmacol. 42, 731–737. 10.1177/009127002401102678 12092740

[B69] Kohlert C. Van Rensen I. März R. Schindler G. Graefe E. U. Veit M. (2000). Bioavailability and Pharmacokinetics of Natural Volatile Terpenes in Animals and Humans. Planta Med. 66, 495–505. 10.1055/s-2000-8616 10985073

[B70] Kon K. V. Rai M. K. (2012). Plant Essential Oils and Their Constituents in Coping with Multidrug-Resistant Bacteria. Expert Rev. Anti Infect. Ther. 10, 775–790. 10.1586/eri.12.57 22943401

[B71] Koyama S. Purk A. Kaur M. Soini H. A. Novotny M. V. Davis K. (2019). Beta-caryophyllene Enhances Wound Healing through Multiple Routes. PLoS one 14, e0216104. 10.1371/journal.pone.0216104 31841509 PMC6913986

[B72] Kunihiro A. G. Luis P. B. Brickey J. A. Frye J. B. Chow H. S. Schneider C. (2019). Beta-Glucuronidase Catalyzes Deconjugation and Activation of Curcumin-Glucuronide in Bone. J. Nat. Prod. 82, 500–509. 10.1021/acs.jnatprod.8b00873 30794412 PMC6528680

[B73] Langat M. K. Mayowa Y. Sadgrove N. Danyaal M. Prescott T. A. K. Kami T. (2021). Multi-layered Antimicrobial Synergism of (E)-caryophyllene with Minor Compounds, Tecleanatalensine B and Normelicopine, from the Leaves of Vepris Gossweileri (I. Verd.) Mziray. Nat. Product. Res., 1–11. 10.1080/14786419.2021.1899176 33719772

[B74] Lassak E. Southwell I. (1974). Occurrence of Some Unusual Compounds in the Leaf Oils of *Eriostemon Obovalis* and *Phebalium Glandulosum* Subsp. *Glandulosum* . Aust. J. Chem. 27, 2703–2705. 10.1071/ch9742703

[B75] Lassila T. Mattila S. Turpeinen M. Pelkonen O. Tolonen A. (2016). Tandem Mass Spectrometric Analysis of S- and N-Linked Glutathione Conjugates of Pulegone and Menthofuran and Identification of P450 Enzymes Mediating Their Formation. Rapid Commun. Mass. Spectrom. 30, 917–926. 10.1002/rcm.7518 26969934

[B76] Lee S. H. Heo Y. Kim Y. C. (2010). Effect of German Chamomile Oil Application on Alleviating Atopic Dermatitis-like Immune Alterations in Mice. J. Vet. Sci. 11, 35–41. 10.4142/jvs.2010.11.1.35 20195063 PMC2833428

[B77] Leonardini A. Laviola L. Perrini S. Natalicchio A. Giorgino F. (2009). Cross-Talk between PPARgamma and Insulin Signaling and Modulation of Insulin Sensitivity. PPAR Res. 2009, 818945. 10.1155/2009/818945 20182551 PMC2826877

[B78] Lesgards J. F. Baldovini N. Vidal N. Pietri S. (2014). Anticancer Activities of Essential Oils Constituents and Synergy with Conventional Therapies: A Review. Phytother Res. 28, 1423–1446. 10.1002/ptr.5165 24831562

[B79] Li J. E. Futawaka K. Yamamoto H. Kasahara M. Tagami T. Liu T. H. (2015). Cinnamaldehyde Contributes to Insulin Sensitivity by Activating PPARδ, PPARγ, and RXR. Am. J. Chin. Med. 43, 879–892. 10.1142/S0192415X15500512 26227398

[B80] Li Y. Fu X. Ma X. Geng S. Jiang X. Huang Q. (2018). Intestinal Microbiome-Metabolome Responses to Essential Oils in Piglets. Front. Microbiol. 9, 1988. 10.3389/fmicb.2018.01988 30210470 PMC6120982

[B81] Liang W. Z. Lu C. H. (2012). Carvacrol-induced [Ca2+]i Rise and Apoptosis in Human Glioblastoma Cells. Life Sci. 90, 703–711. 10.1016/j.lfs.2012.03.027 22480511

[B82] Liu J. Zhu Y. Du G. Zhou J. Chen J. (2013). Response of *Saccharomyces cerevisiae* to *D*-Limonene-Induced Oxidative Stress. Appl. Microbiol. Biotechnol. 97, 6467–6475. 10.1007/s00253-013-4931-9 23644769

[B83] Ma D. He J. He D. (2020). Chamazulene Reverses Osteoarthritic Inflammation through Regulation of Matrix Metalloproteinases (MMPs) and NF-Kβ Pathway in *In-Vitro* and *In-Vivo* Models. Biosci. Biotechnol. Biochem. 84, 402–410. 10.1080/09168451.2019.1682511 31642732

[B84] Mandeville A. Cock I. E. (2018). *Terminalia Chebula* Retz. Fruit Extracts Inhibit Bacterial Triggers of Some Autoimmune Diseases and Potentiate the Activity of Tetracycline. Indian J. Microbiol. 58, 496–506. 10.1007/s12088-018-0754-9 30262960 PMC6141404

[B85] Mao Q. Q. Xu X. Y. Cao S. Y. Gan R. Y. Corke H. Beta T. (2019). Bioactive Compounds and Bioactivities of Ginger (*Zingiber Officinale* Roscoe). Foods 8, 185. 10.3390/foods8060185 PMC6616534 31151279

[B86] Medeiros R. Passos G. F. Vitor C. E. Koepp J. Mazzuco T. L. Pianowski L. F. (2007). Effect of Two Active Compounds Obtained from the Essential Oil of *Cordia Verbenacea* on the Acute Inflammatory Responses Elicited by LPS in the Rat Paw. Br. J. Pharmacol. 151, 618–627. 10.1038/sj.bjp.0707270 17471174 PMC2013990

[B87] Miao Y. Sun Y. Wang W. Du B. Xiao S. E. Hu Y. (2013). 6-Gingerol Inhibits Hair Shaft Growth in Cultured Human Hair Follicles and Modulates Hair Growth in Mice. PLoS one 8, e57226. 10.1371/journal.pone.0057226 23437345 PMC3578824

[B88] Mikulášová M. Chovanová R. Vaverková Š. (2016). Synergism between Antibiotics and Plant Extracts or Essential Oils with Efflux Pump Inhibitory Activity in Coping with Multidrug-Resistant Staphylococci. Phytochemistry Rev. 15, 651–662.

[B89] Miller J. A. Hakim I. A. Chew W. Thompson P. Thomson C. A. Chow H. H. (2010). Adipose Tissue Accumulation of *D*-Limonene with the Consumption of a Lemonade Preparation Rich in *D*-Limonene Content. Nutr. Cancer 62, 783–788. 10.1080/01635581003693066 20661827 PMC6262896

[B90] Mohamed O. I. El-Nahas A. F. El-Sayed Y. S. Ashry K. M. (2016). Ginger Extract Modulates Pb-Induced Hepatic Oxidative Stress and Expression of Antioxidant Gene Transcripts in Rat Liver. Pharm. Biol. 54, 1164–1172. 10.3109/13880209.2015.1057651 26079851

[B91] Moussaieff A. Rimmerman N. Bregman T. Straiker A. Felder C. C. Shoham S. (2008). Incensole Acetate, an Incense Component, Elicits Psychoactivity by Activating TRPV3 Channels in the Brain. FASEB J. 22, 3024–3034. 10.1096/fj.07-101865 18492727 PMC2493463

[B92] Mühlbauer R. C. Lozano A. Palacio S. Reinli A. Felix R. (2003). Common Herbs, Essential Oils, and Monoterpenes Potently Modulate Bone Metabolism. Bone 32, 372–380. 10.1016/s8756-3282(03)00027-9 12689680

[B93] Murni V. W. Saepudin E. Cahyana A. H. Rahayu D. U. C. Hastuti L. T. Haib J. (2016). Effect of Oven Drying and Storage on Essential Oil Composition of Clove (*Syzygium Aromaticum*) from Toli-Toli. Int. Symp. Curr. Prog. Math. Sci. 1862, 030081–030084, .

[B94] Nölder M. Germer S. Koch E. (2011). Pharmacokinetics of Linalool and Linalyl Acetate, the Two Main Constituents of Silexan, an Essential Oil from *Lavandula Angustifolia* Flowers, in Rats. Planta Med. 77, PM44.

[B95] Nsangou M. F. Happi E. N. Fannang S. V. Atangana A. F. Waffo A. F. K. Wansi J. D. (2021). Chemical Composition and Synergistic Antimicrobial Effects of a Vegetatively Propagated Cameroonian Lemon, *Citrus X limon* (L.) Osbeck. ACS Food Sci. Technol. 1, 354–361. 10.1021/acsfoodscitech.0c00071

[B96] Ohland C. L. Macnaughton W. K. (2010). Probiotic Bacteria and Intestinal Epithelial Barrier Function. Am. J. Physiol. Gastrointest. Liver Physiol. 298, G807–G819. 10.1152/ajpgi.00243.2009 20299599

[B97] Okabe H. Obata Y. Takayama K. Nagai T. (1990). Percutaneous Absorption Enhancing Effect and Skin Irritation of Monocyclic Monoterpenes. Drug Des. Deliv. 6, 229–238. 2076182

[B98] Onal E. M. Afsar B. Covic A. Vaziri N. D. Kanbay M. (2019). Gut Microbiota and Inflammation in Chronic Kidney Disease and Their Roles in the Development of Cardiovascular Disease. Hypertens. Res. 42, 123–140. 10.1038/s41440-018-0144-z 30504819

[B99] Passos G. F. Fernandes E. S. Da Cunha F. M. Ferreira J. Pianowski L. F. Campos M. M. (2007). Anti-inflammatory and Anti-allergic Properties of the Essential Oil and Active Compounds from *Cordia Verbenacea* . J. Ethnopharmacol 110, 323–333. 10.1016/j.jep.2006.09.032 17084568

[B100] Pauli A. Schilcher H. (2010). “ *In Vitro* Antimicrobial Activities of Essential Oils Monographed in the European Pharmacopoeia 6th Edition,” in Handbook of Essential Oils: Science, Technology, and Applications. Editors Bașer K. H. C. Buchbauer G. (Boca Raton, FL: CRC Press), 353–547. 10.1201/9781420063165-c12

[B101] Pavan B. Dalpiaz A. Marani L. Beggiato S. Ferraro L. Canistro D. (2018). Geraniol Pharmacokinetics, Bioavailability and its Multiple Effects on the Liver Antioxidant and Xenobiotic-Metabolizing Enzymes. Front. Pharmacol. 9, 18. 10.3389/fphar.2018.00018 29422862 PMC5788896

[B102] Petrie J. R. Guzik T. J. Touyz R. M. (2018). Diabetes, Hypertension, and Cardiovascular Disease: Clinical Insights and Vascular Mechanisms. Can. J. Cardiol. 34, 575–584. 10.1016/j.cjca.2017.12.005 29459239 PMC5953551

[B103] Raman G. Gaikar V. G. (2003). Hydrotropic Solubilization of Boswellic Acids from *Boswellia Serrata* Resin. Langmuir 19, 8026–8032. 10.1021/la034611r

[B104] Russo E. B. (2011). Taming THC: Potential Cannabis Synergy and Phytocannabinoid-Terpenoid Entourage Effects. Br. J. Pharmacol. 163, 1344–1364. 10.1111/j.1476-5381.2011.01238.x 21749363 PMC3165946

[B105] Saad N. Y. Muller C. D. Lobstein A. (2013). Major Bioactivities and Mechanism of Action of Essential Oils and Their Components. Flavour Fragr. J. 28, 269–279. 10.1002/ffj.3165

[B106] Sadgrove N. J. (2020a). Comparing Essential Oils from Australia's 'Victorian Christmas Bush' (Prostanthera Lasianthos Labill., Lamiaceae) to Closely Allied New Species: Phenotypic Plasticity and Taxonomic Variability. Phytochemistry 176, 112403. 10.1016/j.phytochem.2020.112403 32422392

[B107] Sadgrove N. J. Jones G. L. (2013). A Possible Role of Partially Pyrolysed Essential Oils in Australian Aboriginal Traditional Ceremonial and Medicinal Smoking Applications of *Eremophila longifolia* (R. Br.) F. Muell (*Scrophulariaceae*). J. Ethnopharmacol 147, 638–644. 10.1016/j.jep.2013.03.060 23563055

[B108] Sadgrove N. J. Jones G. L. (2014a). Cytogeography of Essential Oil Chemotypes of *Eremophila longifolia* F. Muell (Scrophulariaceae). Phytochemistry 105, 43–51. 10.1016/j.phytochem.2014.05.005 24874947

[B109] Sadgrove N. J. Jones G. L. (2019). From Petri Dish to Patient: Bioavailability Estimation and Mechanism of Action for Antimicrobial and Immunomodulatory Natural Products. Front. Microbiol. 10, 2470. 10.3389/fmicb.2019.02470 31736910 PMC6834656

[B110] Sadgrove N. J. Jones G. L. Greatrex B. W. (2014). Isolation and Characterisation of (-)-genifuranal: The Principal Antimicrobial Component in Traditional Smoking Applications of *Eremophila longifolia* (Scrophulariaceae) by Australian Aboriginal Peoples. J. Ethnopharmacol 154, 758–766. 10.1016/j.jep.2014.05.003 24837304

[B111] Sadgrove N. J. Madeley L. G. Van Wyk B. E. (2019). Volatiles from African Species of *Croton* (Euphorbiaceae), Including New Diterpenes in Essential Oil from *Croton Gratissimus* . Heliyon 5, e02677. 10.1016/j.heliyon.2019.e02677 31687511 PMC6820369

[B112] Sadgrove N. J. Padilla-González G. F. Green A. Langat M. K. Mas-Claret E. Lyddiard D. (2021). The Diversity of Volatile Compounds in Australia's Semi-desert Genus Eremophila (Scrophulariaceae). Plants (Basel) 116, 1170–1178. 10.3390/plants10040785 PMC8073941 33923613

[B113] Sadgrove N. J. Padilla-González G. F. Telford I. R. H. Greatrex B. W. Jones G. L. Andrew R. (2020a). Prostanthera (Lamiaceae) as a 'Cradle of Incense': Chemophenetics of Rare Essential Oils from Both New and Forgotten Australian 'Mint Bush' Species. Plants (Basel) 9, 1570. 10.3390/plants9111570 PMC7696040 33202983

[B114] Sadgrove N. J. Greatrex B. W. Jones G. L. (2015). α-Cyclodextrin Encapsulation Enhances Antimicrobial Activity of Cineole-Rich Essential Oils from Australian Species of Prostanthera (Lamiaceae). Nat. Volatiles Essent. OIls 2, 30–38.

[B115] Sadgrove N. J. Jones G. L. (2014b). Reviewing the Importance of Aromatic Medicinal Plants in the Traditional Pharmacopoeia of Australian Aboriginal People. XXIX International Horticultural Congress on Horticulture: Sustaining Lives, Livelihoods and Landscapes (IHC2014). V World 1125, 297–302.

[B116] Sadgrove N. Jones G. (2015). A Contemporary Introduction to Essential Oils: Chemistry, Bioactivity and Prospects for Australian Agriculture. Agriculture 5, 48–102. 10.3390/agriculture5010048

[B117] Sadgrove N. J. (2020b). Southern Africa as a 'cradle of Incense' in Wider African Aromatherapy. South. Africa as a ‘cradle incense’ wider Scientific Afr. 9, e00502. 10.1016/j.sciaf.2020.e00502

[B118] Sadgrove N. J. Telford I. R. H. Padilla-González G. F. Greatrex B. W. Bruhl J. J. (2020b). GC-MS 'chemophenetics' on Australian Pink-Flowered Phebalium (Rutaceae) Using Herbarium Leaf Material Demonstrates Phenetic Agreement with Putative New Species. Phytochemistry Lett. 38, 112–120. 10.1016/j.phytol.2020.05.014

[B119] Sadgrove N. J. (2021). The 'bald' Phenotype (Androgenetic Alopecia) Is Caused by the High Glycaemic, High Cholesterol and Low mineral 'western Diet'. Trends Food Sci. Tech. 10.1016/j.tifs.2021.06.056

[B120] Sadgrove N. Mijajlovic S. Tucker D. J. Watson K. Jones G. L. (2011). Characterization and Bioactivity of Essential Oils from Novel Chemotypes of *Eremophila longifolia* (F. Muell) (Myoporaceae): a Highly Valued Traditional Australian Medicine. Flavour Fragrance J. 26, 341–350. 10.1002/ffj.2062

[B121] Šadibolová M. Zárybnický T. Smutný T. Pávek P. Šubrt Z. Matoušková P. (2019). Sesquiterpenes Are Agonists of the Pregnane X Receptor but Do Not Induce the Expression of Phase I Drug-Metabolizing Enzymes in the Human Liver. Int. J. Mol. Sci. 20, 4562. 10.3390/ijms20184562 PMC6769599 31540101

[B122] Schnaubelt K. (1999). Medical Aromatherapy: Healing with Essential Oils. Bombay, India: Frog Books.

[B123] Sebai H. Selmi S. Rtibi K. Souli A. Gharbi N. Sakly M. (2013). Lavender (*Lavandula Stoechas* L.) Essential Oils Attenuate Hyperglycemia and Protect against Oxidative Stress in Alloxan-Induced Diabetic Rats. Lipids Health Dis. 12, 189. 10.1186/1476-511X-12-189 24373672 PMC3880178

[B124] Sell C. (2010). “Chapter 5. Chemistry of Essential Oils,” in Handbook of Essential Oils: Science, Technology, and Applications. Editors Başer K. H. C. Buchbauer G. (London: CRC Press, Taylor and Francis Group).

[B125] Serrano E. Cornu A. Kondjoyan N. Figueredo G. Agabriel J. Micol D. (2007). Terpene Accumulation in Muscle and Fatty Tissues of Calves Supplemented with Essential Oils. J. Anim. Feed Sci. 16, 168–179. 10.22358/jafs/66736/2007

[B126] Sharma M. Levenson C. Bell R. H. Anderson S. A. Hudson J. B. Collins C. C. (2014). Suppression of Lipopolysaccharide-Stimulated Cytokine/chemokine Production in Skin Cells by Sandalwood Oils and Purified α-santalol and β-santalol. Phytother Res. 28, 925–932. 10.1002/ptr.5080 24318647

[B127] Sheehan D. Meade G. Foley V. M. Dowd C. A. (2001). Structure, Function and Evolution of Glutathione Transferases: Implications for Classification of Non-mammalian Members of an Ancient Enzyme Superfamily. Biochem. J. 360, 1–16. 10.1042/0264-6021:3600001 11695986 PMC1222196

[B128] Shi F. Zhao Y. Firempong C. K. Xu X. (2016). Preparation, Characterization and Pharmacokinetic Studies of Linalool-Loaded Nanostructured Lipid Carriers. Pharm. Biol. 54, 2320–2328. 10.3109/13880209.2016.1155630 26986932

[B129] Shimoi K. Nakayama T. (2005). Glucuronidase Deconjugation in Inflammation. Methods Enzymol. 400, 263–272. 10.1016/S0076-6879(05)00015-7 16399354

[B130] Smith J. Tucker D. Alter D. Watson K. Jones G. (2010). Intraspecific Variation in Essential Oil Composition of *Eremophila longifolia* F. Muell. (Myoporaceae): Evidence for Three Chemotypes. Phytochemistry 71, 1521–1527. 10.1016/j.phytochem.2010.06.006 20605032

[B131] Soundharrajan I. Kim D. H. Srisesharam S. Kuppusamy P. Choi K. C. (2018). R-limonene Enhances Differentiation and 2-Deoxy-D-Glucose Uptake in 3T3-L1 Preadipocytes by Activating the Akt Signaling Pathway. Evid. Based Complement. Alternat Med. 2018, 4573254. 10.1155/2018/4573254 30250490 PMC6140011

[B132] Sperker B. Tomkiewicz C. Burk O. Barouki R. Kroemer H. K. (2001). Regulation of Human Beta-Glucuronidase by A23187 and Thapsigargin in the Hepatoma Cell Line HepG2. Mol. Pharmacol. 59, 177–182. 10.1124/mol.59.2.177 11160851

[B133] Su Y. C. Cheng T. C. Leu Y. L. Roffler S. R. Wang J. Y. Chuang C. H. (2014). PET Imaging of β-glucuronidase Activity by an Activity-Based 124I-Trapping Probe for the Personalized Glucuronide Prodrug Targeted Therapy. Mol. Cancer Ther. 13, 2852–2863. 10.1158/1535-7163.MCT-14-0212 25277385

[B134] Sueke H. Kaye S. B. Neal T. Hall A. Tuft S. Parry C. M. (2010). An *In Vitro* Investigation of Synergy or Antagonism between Antimicrobial Combinations against Isolates from Bacterial Keratitis. Invest. Ophthalmol. Vis. Sci. 51, 4151–4155. 10.1167/iovs.09-4839 20335613

[B135] Talpur N. Echard B. Ingram C. Bagchi D. Preuss H. (2004). Effects of a Novel Formulation of Essential Oils on Glucose-Insulin Metabolism in Diabetic and Hypertensive Rats: a Pilot Study. Diabetes Obes. Metab. 7, 193–199. 10.1111/j.1463-1326.2004.00386.x 15715893

[B136] Tew K. D. Manevich Y. Grek C. Xiong Y. Uys J. Townsend D. M. (2011). The Role of Glutathione S-Transferase P in Signaling Pathways and S-Glutathionylation in Cancer. Free Radic. Biol. Med. 51, 299–313. 10.1016/j.freeradbiomed.2011.04.013 21558000 PMC3125017

[B137] Thompson D. Constantin-Teodosiu D. Egestad B. Mickos H. Moldéus P. (1990). Formation of Glutathione Conjugates during Oxidation of Eugenol by Microsomal Fractions of Rat Liver and Lung. Biochem. Pharmacol. 39, 1587–1595. 10.1016/0006-2952(90)90525-p 2337416

[B138] Tisserand R. Young R. (2013). Essential Oil Safety: A Guide for Health Care Professionals, 2e. Elsevier.

[B139] Townsend D. M. Tew K. D. (2003). The Role of Glutathione-S-Transferase in Anti-cancer Drug Resistance. Oncogene 22, 7369–7375. 10.1038/sj.onc.1206940 14576844 PMC6361125

[B140] Urasaki Y. Beaumont C. Talbot J. N. Hill D. K. Le T. T. (2020). Akt3 Regulates the Tissue-specific Response to Copaiba Essential Oil. Int. J. Mol. Sci. 21, 2851. 10.3390/ijms21082851 PMC7216139 32325885

[B141] Valdivieso-Ugarte M. Gomez-Llorente C. Plaza-Díaz J. Gil Á. (2019). Antimicrobial, Antioxidant, and Immunomodulatory Properties of Essential Oils: A Systematic Review. Nutrients 11, 2786. 10.3390/nu11112786 PMC6893664 31731683

[B142] Valente A. Carrillo A. E. Tzatzarakis M. N. Vakonaki E. Tsatsakis A. M. Kenny G. P. (2015). The Absorption and Metabolism of a Single L-Menthol Oral versus Skin Administration: Effects on Thermogenesis and Metabolic Rate. Food Chem. Toxicol. 86, 262–273. 10.1016/j.fct.2015.09.018 26429629

[B143] Van Bambeke F. Glupczynski Y. Plésiat P. Pechère J. C. Tulkens P. M. (2003). Antibiotic Efflux Pumps in Prokaryotic Cells: Occurrence, Impact on Resistance and Strategies for the Future of Antimicrobial Therapy. J. Antimicrob. Chemother. 51, 1055–1065. 10.1093/jac/dkg224 12697642

[B144] Van Vuuren S. Holl D. (2017). Antimicrobial Natural Product Research: A Review from a South African Perspective for the Years 2009-2016. J. Ethnopharmacol 208, 236–252. 10.1016/j.jep.2017.07.011 28694104

[B145] Van Vuuren S. Viljoen A. (2011). Plant-based Antimicrobial Studies-Mmethods and Approaches to Study the Interaction between Natural Products. Planta Med. 77, 1168–1182. 10.1055/s-0030-1250736 21283954

[B146] Victor Antony Santiago J. Jayachitra J. Shenbagam M. Nalini N. (2012). Dietary *D*-Limonene Alleviates Insulin Resistance and Oxidative Stress-Induced Liver Injury in High-Fat Diet and L-NAME-Treated Rats. Eur. J. Nutr. 51, 57–68. 10.1007/s00394-011-0182-7 21445622

[B147] Vigushin D. M. Poon G. K. Boddy A. English J. Halbert G. W. Pagonis C. (1998). Phase I and Pharmacokinetic Study of D-Limonene in Patients with Advanced Cancer. Cancer Research Campaign Phase I/II Clinical Trials Committee. Cancer Chemother. Pharmacol. 42, 111–117. 10.1007/s002800050793 9654110

[B148] Wang S. Sun X. Jiang L. Liu X. Chen M. Yao X. (2016). 6-Gingerol Induces Autophagy to Protect HUVECs Survival from Apoptosis. Chem. Biol. Interact 256, 249–256. 10.1016/j.cbi.2016.07.020 27451028

[B149] Wenqiang G. Shufen L. Ruixiang Y. Shaokun T. Can Q. (2007). Comparison of Essential Oils of Clove Buds Extracted with Supercritical Carbon Dioxide and Other Three Traditional Extraction Methods. Food Chem. 101, 1558–1564.

[B150] Wu Y. Zhang Y. Xie G. Zhao A. Pan X. Chen T. (2012). The Metabolic Responses to Aerial Diffusion of Essential Oils. PLoS one 7, e44830. 10.1371/journal.pone.0044830 22984571 PMC3440318

[B151] Xu X. Li Y. Hou J. Zhang S. Xu Y. Wang Y. (2011). Pharmacokinetic Study of Borneol and Menthol in Rats after Oral Administration of Qingyan Drop Pills. Planta Med. 77, 1600–1604. 10.1055/s-0030-1270998 21484670

[B152] Zámboriné Németh É. Thi Nguyen H. (2020). Thujone, a Widely Debated Volatile Compound: what Do We Know about it? Phytochem. Rev. 19, 405–423. 10.1007/s11101-020-09671-y

[B153] Zehetner P. Höferl M. Buchbauer G. (2019). Essential Oil Components and Cytochrome P450 Enzymes: a Review. Flavour Fragr J. 34, 223–240. 10.1002/ffj.3496

[B154] Zhong C. Qu C. Wang B. Liang S. Zeng B. (2017). Probiotics for Preventing and Treating Small Intestinal Bacterial Overgrowth: A Meta-Analysis and Systematic Review of Current Evidence. J. Clin. Gastroenterol. 51, 300–311. 10.1097/MCG.0000000000000814 28267052

[B155] Zhou X. Li J. Guo J. Geng B. Ji W. Zhao Q. (2018). Gut-dependent Microbial Translocation Induces Inflammation and Cardiovascular Events after ST-Elevation Myocardial Infarction. Microbiome 6, 66. 10.1186/s40168-018-0441-4 29615110 PMC5883284

[B156] Zhu R. Liu H. Liu C. Wang L. Ma R. Chen B. (2017). Cinnamaldehyde in Diabetes: A Review of Pharmacology, Pharmacokinetics and Safety. Pharmacol. Res. 122, 78–89. 10.1016/j.phrs.2017.05.019 28559210

[B157] Zidorn C. (2019). Plant Chemophenetics - A New Term for Plant Chemosystematics/plant Chemotaxonomy in the Macro-Molecular Era. Phytochemistry 163, 147–148. 10.1016/j.phytochem.2019.02.013 30846237

